# Supplementary material for: Unequal expression: Social position modulates APOE genotype risk of dementia
Source: PLoS One. 2025 Nov 20;20(11):e0335846. doi: 10.1371/journal.pone.0335846 (PMC12633912; doi:10.1371/journal.pone.0335846)
Supplement: S1 File — (DOCX) [file pone.0335846.s001.docx]

**Supporting information**

**S1. STROBE Statement—Checklist of items that should be included in reports of cohort studies.**

**S2. Social determinants of health and genetic data measurement.**

**S1 Table. Descriptive characteristics of ELSA and HRS sample included at baseline.**

**S2 Table. Analysis of social advantage on the risk of dementia according to APOE allele risk profile.**

**S3 Table. Analysis of social adversity on the risk of dementia according to polygenic risk score of AD profile.**

**S4 Table. Analysis of social adversity on the risk of dementia according to APOE allele risk profile excluding people with mild cognitive impairment.**

**S5 Table. Analysis of social adversity on the risk of dementia according to APOE allele risk profile in dementia cases identified using cognitive and functional impairment only.**

**S6 Table. Analysis of social adversity on the risk of dementia according to APOE allele risk profile adjusting by the number of dementia risk factors.**

**S7 Table. Descriptive statistics of ELSA and HRS sample included and excluded based on complete social determinants of health information available at baseline.**

**S8 Table. Analysis of social adversity on the risk of dementia according to APOE allele risk profile weighting by the inverse probability of being selected in the study.**

**S9 Table. Analysis of social adversity on the risk of dementia according to APOE allele risk profile by sex.**

**S10 Table. Analysis of social adversity on the risk of dementia according to APOE allele risk profile by race.**

**S11 Table. Analysis of social adversity on the risk of dementia according to APOE allele risk profile by individual social determinants.**

**S1 Fig. Participant’s study selection for HRS and ELSA populations.**

**S2 Fig. Social advantage Interacts with APOE Allele to Determine Risk of Developing Dementia.**

**S1. STROBE Statement—Checklist of items that should be included in reports of *cohort studies.***

|  | Item No | Recommendation | Page included |
| --- | --- | --- | --- |
| **Title and abstract** | 1 | (*a*) Indicate the study’s design with a commonly used term in the title or the abstract | 1,2 |
|  |  | (*b*) Provide in the abstract an informative and balanced summary of what was done and what was found | 2 |
| Introduction | | |  |
| Background/rationale | 2 | Explain the scientific background and rationale for the investigation being reported | 3-5 |
| Objectives | 3 | State specific objectives, including any prespecified hypotheses | 5 |
| Methods | | |  |
| Study design | 4 | Present key elements of study design early in the paper | 5 |
| Setting | 5 | Describe the setting, locations, and relevant dates, including periods of recruitment, exposure, follow-up, and data collection | 5 |
| Participants | 6 | (*a*) Give the eligibility criteria, and the sources and methods of selection of participants. Describe methods of follow-up | 6 |
|  |  | (*b*) For matched studies, give matching criteria and number of exposed and unexposed | NA |
| Variables | 7 | Clearly define all outcomes, exposures, predictors, potential confounders, and effect modifiers. Give diagnostic criteria, if applicable | 6-8 |
| Data sources/ measurement | 8* | For each variable of interest, give sources of data and details of methods of assessment (measurement). Describe comparability of assessment methods if there is more than one group | 6-8 |
| Bias | 9 | Describe any efforts to address potential sources of bias | 6-8 |
| Study size | 10 | Explain how the study size was arrived at | 9 |
| Quantitative variables | 11 | Explain how quantitative variables were handled in the analyses. If applicable, describe which groupings were chosen and why | 4-6 |
| Statistical methods | 12 | (*a*) Describe all statistical methods, including those used to control for confounding | 6,7 |
|  |  | (*b*) Describe any methods used to examine subgroups and interactions | 9-10 |
|  |  | (*c*) Explain how missing data were addressed | 6 |
|  |  | (*d*) If applicable, explain how loss to follow-up was addressed | 9-10 |
|  |  | (*e*) Describe any sensitivity analyses | 10-11 |
| Results | | |  |
| Participants | 13* | (a) Report numbers of individuals at each stage of study—eg numbers potentially eligible, examined for eligibility, confirmed eligible, included in the study, completing follow-up, and analysed | 3 |
|  |  | (b) Give reasons for non-participation at each stage | 3 |
|  |  | (c) Consider use of a flow diagram | 3 |
| Descriptive data | 14* | (a) Give characteristics of study participants (eg demographic, clinical, social) and information on exposures and potential confounders | 7 |
|  |  | (b) Indicate number of participants with missing data for each variable of interest | S.1, S.7 |
|  |  | (c) Summarise follow-up time (eg, average and total amount) | 6 |
| Outcome data | 15* | Report numbers of outcome events or summary measures over time | F.1, F.3 |
| Main results | 16 | (*a*) Give unadjusted estimates and, if applicable, confounder-adjusted estimates and their precision (eg, 95% confidence interval). Make clear which confounders were adjusted for and why they were included | T.2, T.3 |
|  |  | (*b*) Report category boundaries when continuous variables were categorized | T.2, T.3 |
|  |  | (*c*) If relevant, consider translating estimates of relative risk into absolute risk for a meaningful time period | NA |
| Other analyses | 17 | Report other analyses done—eg analyses of subgroups and interactions, and sensitivity analyses | 14,15 |
| Discussion | | |  |
| Key results | 18 | Summarise key results with reference to study objectives | 16 |
| Limitations | 19 | Discuss limitations of the study, taking into account sources of potential bias or imprecision. Discuss both direction and magnitude of any potential bias | 19 |
| Interpretation | 20 | Give a cautious overall interpretation of results considering objectives, limitations, multiplicity of analyses, results from similar studies, and other relevant evidence | 16-18 |
| Generalisability | 21 | Discuss the generalisability (external validity) of the study results | 18 |
| Other information | | |  |
| Funding | 22 | Give the source of funding and the role of the funders for the present study and, if applicable, for the original study on which the present article is based | 26 |

*Give information separately for exposed and unexposed groups.

**Note:** An Explanation and Elaboration article discusses each checklist item and gives methodological background and published examples of transparent reporting. The STROBE checklist is best used in conjunction with this article (freely available on the Web sites of PLoS Medicine at http://www.plosmedicine.org/, Annals of Internal Medicine at http://www.annals.org/, and Epidemiology at http://www.epidem.com/). Information on the STROBE Initiative is available at http://www.strobe-statement.org.

**S2. Social determinants of health and genetic data measurement.**

*Social determinants of health measurement*

Education access was measured through educational level divided into three levels for cross-national comparison: less than upper secondary school, upper secondary and vocational, and tertiary education.(1) Economic stability was measured with the mean family income, categorizing people into three groups based on each sample (HRS and ELSA) populational quartile family income: low family income (<25^th^ quartile), intermediate family income (25^th^ to 75^th^ quartile), high family income (>75^th^ quartile).(2) The neighborhood environment was assessed using a measurement scale for perceived neighborhood physical disorder (three items) and social cohesion (four items) with a Likert scale ranging from 1 “Very positive” to 7 “Very negative”;(2) Neighborhood physical disorder and social cohesion were divided into three categories each, according to population quartile scores: low (<25^th^ quartile), intermediate (25^th^ – 75^th^ quartile), and high (>75^th^ quartile). Healthcare access and quality were measured through two questions: a) if people have any type of private healthcare insurance (Yes/No), and b) if they have experienced healthcare discrimination in the last year, understood as ‘a poorer service than other people from doctors or hospitals’(3) (Yes/No). Social context was measured through two variables. The first was mean social support: a Liker format scale ranging from one (“Low support”) to four (“High support”), that assesses perceived children support (six items), spouse/couple support (seven items), and other family members support (seven items).(2) The mean score among children, spouse/couple, and family members' support was calculated and then divided into three categories according to every sample quartile score: low social support (<25^th^ quartile), intermediate (25^th^ to 75^th^ quartile), and high (>75th quartile). The second was the number of discrimination types experienced, divided into three levels for increasing sample size per level: no discrimination experienced, one discrimination type, or two or more types of discrimination experienced.

*Genetic information measurement*

Genetic data was collected in 2006, 2008, and 2010 in the HRS sample. APOE was measured using saliva sample and genotyped using the Illumina Human Omni-2.5-4v1 and Illumina Human Omni-2.5-8v1 Quad BeadChips.(4) Missing call rates of more than 5% and minor allele frequency of less than 5% were used to filter autosomal SNPs. Imputation was performed using the 1000 Genomes Project imputation. European and African genetic ancestry were identified through principal component analysis on independent genome-wide SNPs, with a perfect correlation between genetic ancestry and self-reported race/ethnicity.(5)

Genetic data was collected in 2004-2005 in the ELSA sample. APOE is measured using a blood sample and the Illumina HumanOmni2.5 BeadChips (HumanOmni2.5-4v1, HumanOmni2.5-8v1.3) to genotype the data.(6) Genotyping was performed in two batches. After filtering for 5% of missingness, allele frequency was compared between batches, with correlation overcoming 99%. Imputation was performed using SHAPEIT for pre-phasing, and Minimac3 for imputation using the Haplotype Reference Consortium (HRC.r1-1.GRCh37).(6)

Genetic variants used for identifying APOE allele were rs7412 and rs429358. Individuals of both studies were classified according to their APOE risk profile as low risk (APOE-ε2ε2 or ε2ε3 carriers), intermediate risk (APOE-ε3ε3 carriers), or high risk (APOE-ε2ε4, ε3ε4, or ε4ε4 carriers).

For sensitivity analyses, a polygenic risk score (PGS) for AD dementia that did not include the APOE allele was used. For both samples, the PGS was created using the results from a 2013 GWAS meta-analysis conducted by the International Genomics of Alzheimer’s Project (IGAP), that included 20 independent studies using data from four international cohorts.(7) The methods for the construction of PGS for AD in both samples have been previously described in detail.(8,9) Participants were classified according to their PGS for AD dementia score by cohort and ancestry in three groups: low risk (< 25th quartile score), intermediate risk (25th - 75th quartile score), and high risk (> 75th quartile score) of dementia.

**References**

1. Health Retirement Study. 2017 Spring life history mail survey (LHMS) educational history data. 2021.

2. Survey Research Center UM. Psychosocial and lifestyle questionnaire 2006–2010: Documentation report core section LB. Ann Arbor, MI: Survey Research Center, Institute for Social Research, University of Michigan; 2013.

3. Aravena JM, Chen X, Levy BR. Association between experiencing low healthcare quality and developing dementia. J Am Geriatr Soc. 2024;72(7):2126–32.

4. The 1000 Genomes Project Consortium. A global reference for human genetic variation. Nature. 2015;526(7571):68–74.

5. Bakulski KM, Vadari HS, Faul JD, Heeringa SG, Kardia SLR, Langa KM, et al. Cumulative Genetic Risk and APOE ϵ4 Are Independently Associated With Dementia Status in a Multiethnic, Population-Based Cohort. Neurol Genet. 2021;7(2):e576.

6. English Longitudinal Study of Ageing. Projects: Genetics [Internet]. Available from: https://www.elsa-project.ac.uk/genetics

7. Lambert JC, Ibrahim-Verbaas CA, Harold D, Naj AC, Sims R, Bellenguez C, et al. Meta-analysis of 74,046 individuals identifies 11 new susceptibility loci for Alzheimer’s disease. Nat Genet. 2013;45(12):1452–8.

8. Ajnakina O, Cadar D, Steptoe A. Interplay between Socioeconomic Markers and Polygenic Predisposition on Timing of Dementia Diagnosis. J Am Geriatr Soc. 2020;68(7):1529–36.

9. Ware E, Schmitz L, Faul J, Gard A, Smith, JA, Mitchell, CM, Weir D, Kardia S. Method of construction affects polygenic score prediction of common human traits. bioRxiv. 2017;1–3.

**S1 Table. Descriptive statistics of ELSA and HRS sample included at baseline.**

|  | **ELSA (n: 4,052)** | **HRS (n: 5,797)** | **P-value** |
| --- | --- | --- | --- |
| Follow-up duration in years  Mean, SD (IQR) | 4.86, 2.418 (4 – 8) | 8.27, 3.940 (6 – 12) | 2.2e-16 |
| Age  Mean, SD (IQR) | 67.99, 8.208 (62 – 73) | 68.76, 9.150 (61 – 75) | 2.009e-05 |
| Sex n (%) |  |  |  |
| Male | 1910 (47.1%) | 2494 (43.0%) | 5.304e-05 |
| Female | 2142 (52.9%) | 3303 (57.0%) |  |
| Race/ethnicity n (%) |  |  |  |
| Non-Hispanic White | 4051 (99.9%) | 4781 (82.5%) | NA |
| Non-Hispanic Black | 0 | 766 (13.2%) |  |
| Other (American Indian, Alaskan Native, Asian, and Pacific Islander) | 1 (0.1%) | 250 (4.3%) |  |
| Hispanic | 0 | 454 (7.8%) |  |
| Dementia risk factors n (%) |  |  |  |
| Diabetes | 378 (9.3%) | 1099 (19.0%) | 2.2e-16 |
| Hypertension | 1743 (43.0%) | 3211 (55.4%) | 2.2e-16 |
| Obesity | 1134 (30.9%) | 1825 (31.9%) | 0.3038 |
| High-frequency drinking | 739 (18.6%) | 535 (9.5%) | 2.2e-16 |
| No or little participation in physical activity | 785 (19.4%) | 1467 (25.3%) | 4.356e-12 |
| Smoking | 433 (10.7%) | 717 (12.4%) | 0.007835 |
| Hearing problems | 162 (4.0%) | 273 (4.7%) | 0.09002 |
| Depression | 737 (18.2%) | 1099 (19.0%) | 0.3363 |
| N of dementia risk factors n (%) |  |  |  |
| 0 | 866 (21.4%) | 931 (16.1%) | 2.2e-16 |
| 1 | 1378 (34.0%) | 1753 (30.2%) |  |
| 2 | 999 (24.7%) | 1513 (26.1%) |  |
| ≥3 | 809 (19.9%) | 1600 (27.6%) |  |
| Social determinants of health |  |  |  |
| Education level n (%) |  |  |  |
| Less than upper secondary | 1228 (30.3%) | 984 (17.0%) | 2.2e-16 |
| Upper secondary and vocational | 2085 (51.5%) | 3486 (60.1%) |  |
| Tertiary | 739 (18.2%) | 1327 (22.9%) |  |
| Family income quartile by cohort n (%) |  |  |  |
| <25^th^ quartile income | 902 (22.3%) | 1230 (21.2%) | 0.4651 |
| 25^th^ – 75^th^ quartile income | 2089 (51.6%) | 3031 (52.3%) |  |
| >75^th^ quartile income | 1061 (26.2%) | 1536 (26.5%) |  |
| Healthcare access n (%) |  |  |  |
| No private health insurance | 3519 (86.8%) | 1554 (26.8%) | 2.2e-16 |
| Healthcare discrimination | 168 (4.1%) | 378 (6.5%) | 4.029e-07 |
| Neighborhood environment n (%) |  |  |  |
| Physical disorder |  |  |  |
| Low (<25^th^) | 1341 (33.1%) | 1823 (31.4%) | 1.3e-12 |
| Moderate (25^th^ – 75^th^) | 2005 (49.5%) | 2609 (45.0%) |  |
| High (>75^th^) | 706 (17.4%) | 1365 (23.5%) |  |
| Social cohesion difficulties |  |  |  |
| Low (<25^th^) | 1236 (30.5%) | 1894 (32.7%) | 1.27e-12 |
| Moderate (25^th^ – 75^th^) | 2076 (51.2%) | 2564 (44.2%) |  |
| High (>75^th^) | 740 (18.3%) | 1339 (23.1%) |  |
| Social and community context n (%) |  |  |  |
| Social support |  |  |  |
| Low (<25^th^) | 865 (21.3%) | 1356 (23.4%) | 0.0001346 |
| Moderate (25^th^ – 75^th^) | 2200 (54.3%) | 2897 (50.0%) |  |
| High (>75^th^) | 987 (24.4%) | 1544 (26.6%) |  |
| N of types of discrimination experienced |  |  |  |
| 0 | 2261 (55.8%) | 2431 (41.9%) | 2.2e-16 |
| 1 | 1178 (29.1%) | 2180 (37.6%) |  |
| ≥2 | 613 (15.1%) | 1186 (20.5%) |  |
| N of unfavorable SDH exposed |  |  |  |
| 0 – 1 | 1191 (29.4%) | 3010 (51.9%) | 2.2e-16 |
| 2 | 1465 (36.2%) | 1389 (24.0%) |  |
| 3 | 871 (21.5%) | 795 (13.7%) |  |
| ≥ 4 | 525 (13.0%) | 603 (10.4%) |  |
| APOE allele dosage |  |  |  |
| e2e2 | 20 (0.4%) | 32 (0.6%) | 2.2e-16 |
| e2e3 | 529 (13.1%) | 675 (11.6%) |  |
| e2e4 | 0 | 135 (1.4%) |  |
| e3e3 | 2410 (59.5%) | 3558 (61.4%) |  |
| e3e4 | 916 (22.6%) | 1271 (21.9%) |  |
| e4e4 | 177 (4.4%) | 126 (2.2%) |  |
| Overall APOE dosage |  |  |  |
| e2 (e2e2, e2e3) | 549 (13.5%) | 707 (12.2%) | 0.07765 |
| e3e3 | 2410 (59.5%) | 3558 (61.4%) |  |
| e4 (e2e4, e3e4, e4e4) | 1093 (27.0%) | 1532 (26.4%) |  |
| Any functional limitations in ADL n (%) | 554 (13.7%) | 786 (13.6%) | 0.8715 |

HRS: the Health and Retirement Study; ELSA: the English Longitudinal Study on Aging; SDH: Social Determinants of Health; IQR: interquartile range; NA: not applicable.

<25^th^: less than 25^th^ quartile by cohort; 25^th^ – 75^th^: between 25^th^ and 75^th^ quartile by cohort; >75^th^: more than 75^th^ quartile by cohort.

P-value for chi-square test (X^2^) for nominal and ordinal variables and ANOVA test for continuous variables compariso

| **S2.a Table. Analysis of social advantage on the risk of dementia according to APOE allele risk profile.** | | | | | | | |
| --- | --- | --- | --- | --- | --- | --- | --- |
|  |  | **ELSA** | | **HRS** | | **Fully adjusted Pooled analysis** | **I^2^** |
| **APOE dosage** | **N of favorable SDH** | **N at risk / N event** | **HR (95%CI), p-value** | **N at risk / N event** | **HR (95%CI), p-value** | **HR (95%CI), p-value** |  |
| **e2** | **≤ 1 SDH** | 68/3 | Ref | 42/7 | Ref | Ref |  |
|  | **2 SDH** | 141/7 | 1.17 (0.30 – 4.55) | 149/16 | 0.76 (0.31 – 1.87) | 0.86 (0.41 – 1.81) | 0.0% |
|  | **3 SDH** | 157/6 | 0.66 (0.16 – 2.64) | 169/27 | 0.89 (0.38 – 2.04) | 0.84 (0.41 – 1.71) |  |
|  | **≥ 4 SDH** | 183/6 | 0.70 (0.17 – 2.83) | 347/33 | 0.43 (0.19 – 0.98) * | 0.45 (0.22 – 0.92) * |  |
| **e3e3** | **≤ 1 SDH** | 824/36 | Ref | 225/40 | Ref | Ref |  |
|  | **2 SDH** | 1013/44 | 0.51 (0.28 – 0.93) * | 735/111 | 0.64 (0.44 – 0.92) * | 0.60 (0.44 – 0.82) ** | 0.0% |
|  | **3 SDH** | 453/28 | 0.56 (0.31 – 1.01) | 958/140 | 0.57 (0.40 – 0.80) ** | 0.57 (0.42 – 0.76) *** |  |
|  | **≥ 4 SDH** | 109/5 | 0.60 (0.35 – 1.02) | 1640/220 | 0.45 (0.32 – 0.63) *** | 0.47 (0.35 – 0.62) *** |  |
| **e4** | **≤ 1 SDH** | 156/14 | Ref | 96/25 | Ref | Ref |  |
|  | **2 SDH** | 295/17 | 0.51 (0.25 – 1.05) | 350/61 | 0.61 (0.38 – 0.97) * | 0.59 (0.40 – 0.87) ** | 0.0% |
|  | **3 SDH** | 302/12 | 0.33 (0.15 – 0.72) ** | 417/76 | 0.62 (0.39 – 0.97) * | 0.56 (0.38 – 0.81) ** |  |
|  | **≥ 4 SDH** | 340/26 | 0.49 (0.25 – 0.96) * | 669/124 | 0.53 (0.34 – 0.81) ** | 0.52 (0.37 – 0.75) *** |  |

HRS: the Health and Retirement Study; ELSA: the English Longitudinal Study on Aging; SDH: Social Determinants of Health; HR: Hazard ratio; CI: Confidence Interval; I^2^: Random effect; Ref: reference value.

All models were adjusted by age, sex, and race. The pooled models were additionally adjusted by cohort.

*: p < 0.05; **: p < 0.01; ***: p < 0.001

Social advantage measured as N of favorable SDH exposed: Tertiary education, being in the 75^th^ higher family income range, 75^th^ higher score in neighborhood physical disorder, 75^th^ higher score in neighborhood social cohesion, no experiences of doctors' or hospitals' poorer health service, having some private healthcare insurance, 75^th^ higher score in perceived social support, no experiences of any type of discrimination.

Model properties: all the models' long-rank test p-value < 0.001. Fully adjusted model hazard proportionality assumption based on Schoenfeld residuals. The models meet the hazard proportionality assumption (p-value >0.05). All the variables meet the hazard proportionality assumption (p-value >0.05).

| **S2.b Table. Analysis of social advantage on the risk of dementia according to APOE allele risk profile.** | | | | | | | |
| --- | --- | --- | --- | --- | --- | --- | --- |
|  |  | **ELSA** | | **HRS** | | **Fully adjusted pooled analysis** | **I^2^** |
| **APOE dosage** | **N of favorable SDH** | **N total / N events** | **HR (95%CI), p-value** | **N total / N events** | **HR (95%CI), p-value** | **HR (95%CI), p-value** | 0.0% |
| **e2** | **≤ 1 SDH** | 68/3 | 0.58 (0.17 – 1.94) | 42/7 | 0.72 (0.32 – 1.60) | 0.68 (0.35 – 1.34) |  |
|  | **2 SDH** | 141/7 | 0.73 (0.31 – 1.73) | 149/16 | 0.52 (0.29 – 0.92) * | 0.57 (0.35 – 0.91) * |  |
|  | **3 SDH** | 157/6 | 0.40 (0.16 – 0.99) * | 169/27 | 0.60 (0.37 – 0.98) * | 0.55 (0.36 – 0.85) ** |  |
|  | **≥ 4 SDH** | 183/6 | 0.38 (0.15 – 0.94) * | 347/33 | 0.28 (0.18 – 0.45) *** | 0.29 (0.19 – 0.43) *** |  |
| **e3e3** | **≤ 1 SDH** | 824/36 | Ref | 225/40 | Ref | Ref |  |
|  | **2 SDH** | 1013/44 | 0.51 (0.28 – 0.93) * | 735/111 | 0.65 (0.45 – 0.93) * | 0.61 (0.45 – 0.83) ** |  |
|  | **3 SDH** | 453/28 | 0.56 (0.31 – 1.00) | 958/140 | 0.57 (0.40 – 0.81) ** | 0.56 (0.42 – 0.76) *** |  |
|  | **≥ 4 SDH** | 109/5 | 0.60 (0.35 – 1.02) | 1640/220 | 0.45 (0.32 – 0.63) *** | 0.47 (0.35 – 0.62) *** |  |
| **e4** | **≤ 1 SDH** | 156/14 | 1.61 (0.81 – 3.19) | 96/25 | 1.29 (0.78 – 2.12) | 1.34 (0.90 – 2.01) |  |
|  | **2 SDH** | 295/17 | 0.89 (0.46 – 1.70) | 350/61 | 0.81 (0.54 – 1.20) | 0.81 (0.58 – 1.14) |  |
|  | **3 SDH** | 302/12 | 0.59 (0.29 – 1.21) | 417/76 | 0.82 (0.56 – 1.21) | 0.77 (0.55 – 1.07) |  |
|  | **≥ 4 SDH** | 340/26 | 0.81 (0.45 – 1.45) | 669/124 | 0.73 (0.51 – 1.05) | 0.75 (0.55 – 1.01) |  |

HRS: the Health and Retirement Study; ELSA: the English Longitudinal Study on Aging; SDH: Social Determinants of Health; HR: Hazard ratio; CI: Confidence Interval; I^2^: Random effect; Ref: reference value.

All models were adjusted by age, sex, and race. The pooled models were additionally adjusted by cohort.

*: p < 0.05; **: p < 0.01; ***: p < 0.001

Social advantage measured as N of favorable SDH exposed: Tertiary education, being in the 75^th^ higher family income range, 75^th^ higher score in neighborhood physical disorder, 75^th^ higher score in neighborhood social cohesion, no experiences of doctors' or hospitals' poorer health service, having some private healthcare insurance, 75^th^ higher score in perceived social support, no experiences of any type of discrimination.

Model properties: all the models' long-rank test p-value < 0.001. Fully adjusted model hazard proportionality assumption based on Schoenfeld residuals. The models meet the hazard proportionality assumption (p-value >0.05). All the variables meet the hazard proportionality assumption (p-value >0.05).

| **S3.a Table. Analysis of social adversity on the risk of dementia according to polygenic risk score of AD profile.** | | | | | | | |
| --- | --- | --- | --- | --- | --- | --- | --- |
|  |  | **ELSA** | | **HRS** | | **Fully adjusted Pooled analysis** | **I^2^** |
| **Polygenic risk score** | **N of SDH** | **N at risk / N event** | **HR (95%CI), p-value** | **N at risk / N event** | **HR (95%CI), p-value** | **HR (95%CI), p-value** |  |
| **< 25^th^** | **≤ 1 SDH** | 286/6 | Ref | 734/98 | Ref | Ref |  |
|  | **2 SDH** | 365/11 | 1.32 (0.49 – 3.59) | 310/39 | 1.00 (0.68 – 1.45) | 1.04 (0.73 – 1.46) | 0.0% |
|  | **3 SDH** | 238/9 | 2.13 (0.76 – 5.99) | 150/30 | 1.92 (1.25 – 2.94) ** | 1.91 (1.29 – 3.81) ** |  |
|  | **≥ 4 SDH** | 141/11 | 5.82 (2.14 – 15.88) *** | 97/21 | 2.40 (1.43 – 4.06) ** | 2.96 (1.91 – 4.60) *** |  |
| **25^th^ – 75^th^** | **≤ 1 SDH** | 619/30 | Ref | 1346/168 | Ref | Ref |  |
|  | **2 SDH** | 713/33 | 1.11 (0.67 – 1.83) | 571/75 | 1.16 (0.88 – 1.52) | 1.16 (0.93 – 1.47) | 0.0% |
|  | **3 SDH** | 427/26 | 1.52 (0.89 – 2.61) | 300/56 | 2.16 (1.59 – 2.93) *** | 1.98 (1.52 – 2.58) *** |  |
|  | **≥ 4 SDH** | 248/18 | 2.32 (1.29 – 4.19) ** | 224/50 | 2.82 (2.03 – 3.91) *** | 2.69 (2.02 – 3.58) *** |  |
| **>75^th^** | **≤ 1 SDH** | 286/9 | Ref | 636/85 | Ref | Ref |  |
|  | **2 SDH** | 387/22 | 1.86 (0.84 – 4.01) | 278/49 | 1.83 (1.28 – 2.63) *** | 1.73 (1.25 – 2.40) *** | 0.0% |
|  | **3 SDH** | 206/17 | 3.43 (1.52 – 7.72) ** | 162/27 | 1.84 (1.18 – 2.88) ** | 2.14 (1.47 – 3.11) *** |  |
|  | **≥ 4 SDH** | 136/13 | 4.73 (2.00 – 11.17) *** | 108/23 | 2.32 (1.39 – 3.86) ** | 2.88 (1.89 – 4.39) *** |  |
| **P for interaction** | |  | 0.2511 |  | 0.268 | 0.533 |  |

HRS: the Health and Retirement Study; ELSA: the English Longitudinal Study on Aging; SDH: Social Determinants of Health; HR: Hazard ratio; CI: Confidence Interval; I^2^: Random effect; Ref: reference value.

All models were adjusted by age, sex, and race. The pooled models were additionally adjusted by cohort.

*: p < 0.05; **: p < 0.01; ***: p < 0.001

Social adversity measured as N of unfavorable SDH exposed: Less than upper secondary education, being in the 25^th^ lower family income range, 25^th^ lower score in neighborhood physical disorder, 25^th^ lower score in neighborhood social cohesion, having experienced poorer health service from a doctor or hospital, not having some private healthcare insurance, 25^th^ lower score in perceived social support, having experienced two or more different types of discrimination.

Model properties: all the models' long-rank test p-value < 0.001. Fully adjusted model hazard proportionality assumption based on Schoenfeld residuals. The models meet the hazard proportionality assumption (p-value >0.05). All the variables meet the hazard proportionality assumption (p-value >0.05).

| **S3.b Table. Analysis of social adversity on the risk of dementia according to polygenic risk score of AD profile.** | | | | | | | |
| --- | --- | --- | --- | --- | --- | --- | --- |
|  |  | **ELSA** | | **HRS** | | **Fully adjusted pooled analysis** | **I^2^** |
| **Polygenic score** | **N of SDH** | **N total / N events** | **HR (95%CI), p-value** | **N total / N events** | **HR (95%CI), p-value** | **HR (95%CI), p-value** | 0.0% |
| **< 25^th^** | **≤ 1 SDH** | 286/6 | 0.49 (0.20 – 1.18) | 734/98 | 1.07 (0.84 – 1.38) | 0.99 (0.78 – 1.26) |  |
|  | **2 SDH** | 365/11 | 0.69 (0.35 – 1.38) | 310/39 | 1.04 (0.73 – 1.47) | 0.96 (0.70 – 1.31) |  |
|  | **3 SDH** | 238/9 | 1.09 (0.52 – 2.31) | 150/30 | 2.02 (1.37 – 2.99) *** | 1.75 (1.23 – 2.47) ** |  |
|  | **≥ 4 SDH** | 141/11 | 2.78 (1.39 – 5.58) ** | 97/21 | 2.48 (1.56 – 3.95) *** | 2.64 (1.81 – 3.87) *** |  |
| **25^th^ – 75^th^** | **≤ 1 SDH** | 619/30 | Ref | 1346/168 | Ref | Ref |  |
|  | **2 SDH** | 713/33 | 1.13 (0.69 – 1.85) | 571/75 | 1.15 (0.88 – 1.51) | 1.17 (0.92 – 1.48) |  |
|  | **3 SDH** | 427/26 | 1.54 (0.91 – 2.62) | 300/56 | 2.14 (1.58 – 2.91) ** | 1.99 (1.53 – 2.59) *** |  |
|  | **≥ 4 SDH** | 248/18 | 2.24 (1.25 – 4.02) ** | 224/50 | 2.74 (1.98 – 3.78) *** | 2.66 (2.01 – 3.53) *** |  |
| **>75^th^** | **≤ 1 SDH** | 286/9 | 0.71 (0.34 – 1.51) | 636/85 | 0.98 (0.75 – 1.27) | 0.94 (0.73 – 1.20) |  |
|  | **2 SDH** | 387/22 | 1.25 (0.72 – 2.18) | 278/49 | 1.87 (1.36 – 2.57) *** | 1.72 (1.31 – 2.26) *** |  |
|  | **3 SDH** | 206/17 | 2.48 (1.37 – 4.51) ** | 162/27 | 1.88 (1.25 – 2.82) ** | 2.11 (1.52 – 2.94) *** |  |
|  | **≥ 4 SDH** | 136/13 | 3.68 (1.91 – 7.08) *** | 108/23 | 2.59 (1.66 – 4.04) *** | 2.98 (2.08 – 4.29) *** |  |

HRS: the Health and Retirement Study; ELSA: the English Longitudinal Study on Aging; SDH: Social Determinants of Health; HR: Hazard ratio; CI: Confidence Interval; I^2^: Random effect; Ref: reference value.

All models were adjusted by age, sex, and race. The pooled models were additionally adjusted by cohort.

*: p < 0.05; **: p < 0.01; ***: p < 0.001

Social adversity measured as N of unfavorable SDH exposed: Less than upper secondary education, being in the 25^th^ lower family income range, 25^th^ lower score in neighborhood physical disorder, 25^th^ lower score in neighborhood social cohesion, having experienced poorer health service from a doctor or hospital, not having some private healthcare insurance, 25^th^ lower score in perceived social support, having experienced two or more different types of discrimination.

Model properties: all the models' long-rank test p-value < 0.001. Fully adjusted model hazard proportionality assumption based on Schoenfeld residuals. The models meet the hazard proportionality assumption (p-value >0.05). All the variables meet the hazard proportionality assumption (p-value >0.05).

| **S4.a Table. Analysis of social adversity on the risk of dementia according to APOE allele risk profile excluding people with mild cognitive impairment.** | | | | | | | |
| --- | --- | --- | --- | --- | --- | --- | --- |
|  |  | **ELSA (n: 3,852)** | | **HRS (n: 5,503)** | | **Fully adjusted Pooled analysis** | **I^2^** |
| **APOE dosage** | **N of SDH** | **N at risk / N event** | **HR (95%CI), p-value** | **N at risk / N event** | **HR (95%CI), p-value** | **HR (95%CI), p-value** |  |
| **e2** | **≤ 1 SDH** | 167/2 | Ref | 394/32 | Ref | Ref |  |
|  | **2 SDH** | 174/7 | 4.06 (0.84 – 19.64) | 149/16 | 1.38 (0.75 – 2.55) | 1.62 (0.94 – 2.78) | 0.0% |
|  | **3 SDH** | 105/7 | 6.98 (1.45 – 33.70) * | 67/9 | 2.14 (1.02 – 4.50) * | 2.72 (1.48 – 4.99) ** |  |
|  | **≥ 4 SDH** | 79/3 | 5.10 (0.84 – 31.05) | 57/12 | 5.88 (2.90 – 11.96) *** | 4.90 (2.59 – 9.28) *** |  |
| **e3e3** | **≤ 1 SDH** | 685/23 | Ref | 1805/205 | Ref | Ref |  |
|  | **2 SDH** | 839/25 | 0.91 (0.52 – 1.61) | 817/99 | 1.25 (0.98 – 1.59) | 1.19 (0.95 – 1.49) | 0.0% |
|  | **3 SDH** | 501/21 | 1.69 (0.93 – 3.08) | 448/80 | 2.18 (1.68 – 2.84) *** | 2.10 (1.65 – 2.68) *** |  |
|  | **≥ 4 SDH** | 281/18 | 3.14 (1.69 – 5.85) *** | 333/62 | 2.77 (2.06 – 3.73) *** | 2.88 (2.21 – 3.76) *** |  |
| **e4** | **≤ 1 SDH** | 301/14 | Ref | 731/119 | Ref | Ref |  |
|  | **2 SDH** | 376/18 | 1.32 (0.65 – 2.67) | 353/61 | 1.21 (0.89 – 1.65) | 1.20 (0.91 – 1.59) | 0.0% |
|  | **3 SDH** | 214/15 | 1.70 (0.82 – 3.52) | 211/43 | 1.81 (1.27 – 2.57) ** | 1.75 (1.28 – 2.40) *** |  |
|  | **≥ 4 SDH** | 130/15 | 3.73 (1.79 – 7.78) *** | 138/25 | 1.68 (1.06 – 2.65) * | 2.09 (1.44 – 3.04) *** |  |
| **P for interaction** | |  | 0.7358 |  | 0.0026 | 0.00467 |  |

HRS: the Health and Retirement Study; ELSA: the English Longitudinal Study on Aging; SDH: Social Determinants of Health; HR: Hazard ratio; CI: Confidence Interval; I^2^: Random effect; Ref: reference value.

All models were adjusted by age, sex, and race. The pooled models were additionally adjusted by cohort.

*: p < 0.05; **: p < 0.01; ***: p < 0.001

Mild cognitive impairment defines as

Social adversity measured as N of unfavorable SDH exposed: Less than upper secondary education, being in the 25^th^ lower family income range, 25^th^ lower score in neighborhood physical disorder, 25^th^ lower score in neighborhood social cohesion, having experienced poorer health service from a doctor or hospital, not having some private healthcare insurance, 25^th^ lower score in perceived social support, having experienced two or more different types of discrimination.

Model properties: all the models' long-rank test p-value < 0.001. Fully adjusted model hazard proportionality assumption based on Schoenfeld residuals. The models meet the hazard proportionality assumption (p-value >0.05). All the variables meet the hazard proportionality assumption (p-value >0.05).

| **S4.b Table. Analysis of social adversity on the risk of dementia according to APOE allele risk profile excluding people with mild cognitive impairment.** | | | | | | | |
| --- | --- | --- | --- | --- | --- | --- | --- |
|  |  | **ELSA (n: 3,852)** | | **HRS (n: 5,503)** | | **Fully adjusted pooled analysis** | **I^2^** |
| **APOE dosage** | **N of SDH** | **N total / N events** | **HR (95%CI), p-value** | **N total / N events** | **HR (95%CI), p-value** | **HR (95%CI), p-value** | 0.0% |
| **e2** | **≤ 1 SDH** | 167/2 | 0.32 (0.07 – 1.35) | 394/32 | 0.68 (0.47 – 0.98) * | 0.63 (0.44 – 0.91) * |  |
|  | **2 SDH** | 174/7 | 1.25 (0.54 – 2.92) | 149/16 | 0.97 (0.58 – 1.62) | 1.06 (0.69 – 1.63) |  |
|  | **3 SDH** | 105/7 | 2.08 (0.89 – 4.86) | 67/9 | 1.47 (0.75 – 2.87) | 1.74 (1.05 – 2.89) * |  |
|  | **≥ 4 SDH** | 79/3 | 1.61 (0.48 – 5.37) | 57/12 | 4.24 (2.36 – 7.62) *** | 3.38 (2.00 – 5.73) *** |  |
| **e3e3** | **≤ 1 SDH** | 685/23 | Ref | 1805/205 | Ref | Ref |  |
|  | **2 SDH** | 839/25 | 0.94 (0.53 – 1.66) | 817/99 | 1.25 (0.99 – 1.60) | 1.20 (0.96 – 1.49) |  |
|  | **3 SDH** | 501/21 | 1.76 (0.97 – 3.19) | 448/80 | 2.19 (1.69 – 2.84) *** | 2.10 (1.66 – 2.67) *** |  |
|  | **≥ 4 SDH** | 281/18 | 3.16 (1.70 – 5.87) *** | 333/62 | 2.80 (2.10 – 3.75) *** | 2.91 (2.24 – 3.78) *** |  |
| **e4** | **≤ 1 SDH** | 301/14 | 1.45 (0.75 – 2.83) | 731/119 | 1.70 (1.35 – 2.13) *** | 1.68 (1.35 – 2.08) *** |  |
|  | **2 SDH** | 376/18 | 1.91 (1.03 – 3.55) * | 353/61 | 2.01 (1.51 – 2.68) *** | 1.99 (1.54 – 2.58) *** |  |
|  | **3 SDH** | 214/15 | 2.38 (1.24 – 4.56) ** | 211/43 | 2.98 (2.14 – 4.16) *** | 2.86 (2.14 – 3.84) *** |  |
|  | **≥ 4 SDH** | 130/15 | 5.11 (2.66 – 9.81) *** | 138/25 | 2.64 (1.72 – 4.05) *** | 3.27 (2.32 – 4.63) *** |  |

HRS: the Health and Retirement Study; ELSA: the English Longitudinal Study on Aging; SDH: Social Determinants of Health; HR: Hazard ratio; CI: Confidence Interval; I^2^: Random effect; Ref: reference value.

All models were adjusted by age, sex, and race. The pooled models were additionally adjusted by cohort.

*: p < 0.05; **: p < 0.01; ***: p < 0.001

Mild cognitive impairment defined as

Social adversity measured as N of unfavorable SDH exposed: Less than upper secondary education, being in the 25^th^ lower family income range, 25^th^ lower score in neighborhood physical disorder, 25^th^ lower score in neighborhood social cohesion, having experienced poorer health service from a doctor or hospital, not having some private healthcare insurance, 25^th^ lower score in perceived social support, having experienced two or more different types of discrimination.

Model properties: all the models' long-rank test p-value < 0.001. Fully adjusted model hazard proportionality assumption based on Schoenfeld residuals. The models meet the hazard proportionality assumption (p-value >0.05). All the variables meet the hazard proportionality assumption (p-value >0.05).

| **S5.a Table. Analysis of social adversity on the risk of dementia according to APOE allele risk profile in dementia cases identified using cognitive and functional impairment only.** | | | | | | | |
| --- | --- | --- | --- | --- | --- | --- | --- |
|  |  | **ELSA (n: 4,051)** | | **HRS (n: 5,792)** | | **Fully adjusted Pooled analysis** | **I^2^** |
| **APOE dosage** | **N of SDH** | **N at risk / N event** | **HR (95%CI), p-value** | **N at risk / N event** | **HR (95%CI), p-value** | **HR (95%CI), p-value** |  |
| **e2** | **≤ 1 SDH** | 168/2 | Ref | 403/33 | Ref | Ref |  |
|  | **2 SDH** | 186/6 | 3.17 (0.64 – 15.79) | 158/13 | 1.04 (0.54 – 2.00) | 1.24 (0.70 – 2.19) | 0.0% |
|  | **3 SDH** | 113/8 | 7.74 (1.64 – 36.56) ** | 77/12 | 2.48 (1.27 – 4.84) ** | 2.68 (1.48 – 4.87) *** |  |
|  | **≥ 4 SDH** | 82/3 | 5.26 (0.86 – 32.10) | 69/16 | 5.17 (2.68 – 9.95) *** | 4.60 (2.53 – 8.35) *** |  |
| **e3e3** | **≤ 1 SDH** | 703/15 | Ref | 1845/181 | Ref | Ref |  |
|  | **2 SDH** | 878/25 | 1.29 (0.68 – 2.44) | 859/97 | 1.31 (1.02 – 1.69) * | 1.30 (1.03 – 1.64) * | 0.0% |
|  | **3 SDH** | 527/21 | 2.20 (1.13 – 4.29) * | 477/71 | 1.99 (1.51 – 2.64) *** | 2.03 (1.58 – 2.62) *** |  |
|  | **≥ 4 SDH** | 301/20 | 4.84 (2.47 – 9.48) *** | 375/77 | 3.38 (2.55 – 4.48) *** | 3.60 (2.78 – 4.66) *** |  |
| **e4** | **≤ 1 SDH** | 319/7 | Ref | 762/91 | Ref | Ref |  |
|  | **2 SDH** | 401/11 | 1.43 (0.55 – 3.72) | 368/48 | 1.20 (0.84 – 1.70) | 1.21 (0.87 – 1.68) | 0.0% |
|  | **3 SDH** | 231/8 | 1.72 (0.62 – 4.77) | 240/45 | 2.12 (1.47 – 3.05) *** | 2.01 (1.43 – 2.83) *** |  |
|  | **≥ 4 SDH** | 142/11 | 4.87 (1.87 – 12.68) ** | 159/29 | 2.14 (1.37 – 3.34) *** | 2.53 (1.71 – 3.75) *** |  |
| **P for interaction** | |  | 0.944 |  | 0.0110 | 0.009334 |  |

HRS: the Health and Retirement Study; ELSA: the English Longitudinal Study on Aging; SDH: Social Determinants of Health; HR: Hazard ratio; CI: Confidence Interval; I^2^: Random effect; Ref: reference value.

All models were adjusted by age, sex, and race. The pooled models were additionally adjusted by cohort.

*: p < 0.05; **: p < 0.01; ***: p < 0.001

Dementia defined according to

Social adversity measured as N of unfavorable SDH exposed: Less than upper secondary education, being in the 25^th^ lower family income range, 25^th^ lower score in neighborhood physical disorder, 25^th^ lower score in neighborhood social cohesion, having experienced poorer health service from a doctor or hospital, not having some private healthcare insurance, 25^th^ lower score in perceived social support, having experienced two or more different types of discrimination.

Model properties: all the models' long-rank test p-value < 0.001. Fully adjusted model hazard proportionality assumption based on Schoenfeld residuals. The models meet the hazard proportionality assumption (p-value >0.05). All the variables meet the hazard proportionality assumption (p-value >0.05).

| **S5.b Table. Analysis of social adversity on the risk of dementia according to APOE allele risk profile in dementia cases identified using cognitive and functional impairment only.** | | | | | | | |
| --- | --- | --- | --- | --- | --- | --- | --- |
|  |  | **ELSA** | | **HRS** | | **Fully adjusted pooled analysis** | **I^2^** |
| **APOE dosage** | **N of SDH** | **N total / N events** | **HR (95%CI), p-value** | **N total / N events** | **HR (95%CI), p-value** | **HR (95%CI), p-value** | 0.0% |
| **e2** | **0 SDH** | 168/2 | 0.52 (0.12 – 2.27) | 403/33 | 0.77 (0.53 – 1.12) | 0.75 (0.52 – 1.08) |  |
|  | **1 SDH** | 186/6 | 1.48 (0.58 – 3.83) | 158/13 | 0.85 (0.48 – 1.50) | 0.98 (0.61 – 1.57) |  |
|  | **2 SDH** | 113/8 | 3.64 (1.54 – 8.60) | 77/12 | 2.04 (1.14 – 3.67) * | 2.45 (1.54 – 3.89) *** |  |
|  | **≥ 3SDH** | 82/3 | 2.19 (0.63 – 7.58) | 69/16 | 4.67 (2.78 – 7.84) *** | 4.04 (2.51 – 6.51) *** |  |
| **e3e3** | **0 SDH** | 703/15 | Ref | 1845/181 | Ref | Ref |  |
|  | **1 SDH** | 878/25 | 1.32 (0.70 – 2.51) | 859/97 | 1.32 (1.03 – 1.69) * | 1.32 (1.05 – 1.66) * |  |
|  | **2 SDH** | 527/21 | 2.31 (1.19 – 4.49) * | 477/71 | 1.99 (1.51 – 2.63) *** | 2.05 (1.59 – 2.63) *** |  |
|  | **≥ 3SDH** | 301/20 | 4.84 (2.47 – 9.46) *** | 375/77 | 3.40 (2.58 – 4.48) *** | 3.63 (2.82 – 4.67) *** |  |
| **e4** | **0 SDH** | 319/7 | 1.09 (0.45 – 2.68) | 762/91 | 1.43 (1.11 – 1.84) ** | 1.41 (1.10 – 1.79) ** |  |
|  | **1 SDH** | 401/11 | 1.57 (0.72 – 3.43) | 368/48 | 1.68 (1.22 – 2.31) ** | 1.63 (1.22 – 2.19) ** |  |
|  | **2 SDH** | 231/8 | 1.84 (0.78 – 4.35) | 240/45 | 2.94 (2.11 – 4.09) *** | 2.67 (1.97 – 3.64) *** |  |
|  | **≥ 3SDH** | 142/11 | 5.14 (2.36 – 11.22) *** | 159/29 | 2.90 (1.93 – 4.34) *** | 3.32 (2.34 – 4.72) *** |  |

HRS: the Health and Retirement Study; ELSA: the English Longitudinal Study on Aging; SDH: Social Determinants of Health; HR: Hazard ratio; CI: Confidence Interval; I^2^: Random effect; Ref: reference value.

All models were adjusted by age, sex, and race. The pooled models were additionally adjusted by cohort.

*: p < 0.05; **: p < 0.01; ***: p < 0.001

Dementia defined according to

Social adversity measured as N of unfavorable SDH exposed: Less than upper secondary education, being in the 25^th^ lower family income range, 25^th^ lower score in neighborhood physical disorder, 25^th^ lower score in neighborhood social cohesion, having experienced poorer health service from a doctor or hospital, not having some private healthcare insurance, 25^th^ lower score in perceived social support, having experienced two or more different types of discrimination.

Model properties: all the models' long-rank test p-value < 0.001. Fully adjusted model hazard proportionality assumption based on Schoenfeld residuals. The models meet the hazard proportionality assumption (p-value >0.05). All the variables meet the hazard proportionality assumption (p-value >0.05).

| **S6.a Table. Analysis of social adversity on the risk of dementia according to APOE allele risk profile adjusting by the number of dementia risk factors.** | | | | | | | |
| --- | --- | --- | --- | --- | --- | --- | --- |
|  |  | **ELSA** | | **HRS** | | **Fully adjusted Pooled analysis** | **I^2^** |
| **APOE dosage** | **N of SDH** | **N at risk / N event** | **HR (95%CI), p-value** | **N at risk / N event** | **HR (95%CI), p-value** | **HR (95%CI), p-value** |  |
| **e2** | **≤ 1 SDH** | 168/2 | Ref | 403/37 | Ref | Ref |  |
|  | **2 SDH** | 186/8 | 3.59 (0.75 – 17.23) | 158/18 | 1.30 (0.73 – 2.30) | 1.50 (0.90 – 2.49) | 0.0% |
|  | **3 SDH** | 113/8 | 7.02 (1.48 – 33.23) * | 77/12 | 2.19 (1.13 – 4.22) * | 2.65 (1.53 – 4.61) *** |  |
|  | **≥ 4 SDH** | 82/4 | 4.90 (0.85 – 28.43) | 69/16 | 3.96 (2.08 – 7.52) *** | 3.59 (2.00 – 6.44) *** |  |
| **e3e3** | **≤ 1 SDH** | 173/8 | Ref | 1845/222 | Ref | Ref |  |
|  | **2 SDH** | 821/25 | 1.00 (0.60 – 1.65) | 861/118 | 1.24 (0.99 – 1.55) | 1.20 (0.98 – 1.47) | 0.0% |
|  | **3 SDH** | 823/34 | 1.60 (0.93 – 2.73) | 477/89 | 1.86 (1.44 – 2.40) *** | 1.82 (1.45 – 2.28) *** |  |
|  | **≥ 4 SDH** | 593/47 | 2.65 (1.50 – 4.68) *** | 375/82 | 2.64 (2.02 – 3.46) *** | 2.67 (2.09 – 3.40) *** |  |
| **e4** | **≤ 1 SDH** | 319/16 | Ref | 762/132 | Ref | Ref |  |
|  | **2 SDH** | 401/22 | 1.23 (0.64 – 2.37) | 370/67 | 1.17 (0.87 – 1.58) | 1.17 (0.90 – 1.53) | 0.0% |
|  | **3 SDH** | 231/16 | 1.52 (0.76 – 3.06) | 241/54 | 1.71 (1.23 – 2.36) ** | 1.64 (1.22 – 2.19) *** |  |
|  | **≥ 4 SDH** | 142/15 | 2.62 (1.27 – 5.40) ** | 159/33 | 1.69 (1.12 – 2.54) * | 1.88 (1.33 – 2.66) *** |  |
| **P for interaction** | |  | 0.4859 |  | 0.00144 | 0.00102 |  |

HRS: the Health and Retirement Study; ELSA: the English Longitudinal Study on Aging; SDH: Social Determinants of Health; HR: Hazard ratio; CI: Confidence Interval; I^2^: Random effect; Ref: reference value.

All models were adjusted by age, sex, and race. The pooled models were additionally adjusted by cohort.

*: p < 0.05; **: p < 0.01; ***: p < 0.001

Social adversity measured as N of unfavorable SDH exposed: Less than upper secondary education, being in the 25^th^ lower family income range, 25^th^ lower score in neighborhood physical disorder, 25^th^ lower score in neighborhood social cohesion, having experienced poorer health service from a doctor or hospital, not having some private healthcare insurance, 25^th^ lower score in perceived social support, having experienced two or more different types of discrimination.

Dementia risk factors assessed: hypertension, diabetes, obesity, smoking, high-frequency drinking (drinking more than 5 days a week), physical inactivity (participating <1 time per week in moderate or vigorous physical activity), self-reported hearing problems, and depression (CES-D score >2 points).

Model properties: all the models' long-rank test p-value < 0.001. Fully adjusted model hazard proportionality assumption based on Schoenfeld residuals. The models meet the hazard proportionality assumption (p-value >0.05). All the variables meet the hazard proportionality assumption (p-value >0.05).

| **S6.b Table. Analysis of social adversity on the risk of dementia according to APOE allele risk profile adjusting by the number of dementia risk factors.** | | | | | | | |
| --- | --- | --- | --- | --- | --- | --- | --- |
|  |  | **ELSA** | | **HRS** | | **Fully adjusted pooled analysis** | **I^2^** |
| **APOE dosage** | **N of SDH** | **N total / N events** | **HR (95%CI), p-value** | **N total / N events** | **HR (95%CI), p-value** | **HR (95%CI), p-value** | 0.0% |
| **e2** | **≤ 1 SDH** | 168/2 | 0.30 (0.07 – 1.26) | 403/37 | 0.71 (0.50 – 1.01) | 0.66 (0.47 – 0.93) * |  |
|  | **2 SDH** | 186/8 | 1.14 (0.52 – 2.51) | 158/18 | 0.96 (0.60 – 1.56) | 1.03 (0.69 – 1.55) |  |
|  | **3 SDH** | 113/8 | 2.04 (0.92 – 4.48) | 77/12 | 1.62 (0.91 – 2.90) | 1.81 (1.14 – 2.85) * |  |
|  | **≥ 4 SDH** | 82/4 | 1.39 (0.48 – 4.01) | 69/16 | 3.21 (1.92 – 5.37) *** | 2.67 (1.68 – 4.23) *** |  |
| **e3e3** | **≤ 1 SDH** | 173/8 | Ref | 1845/222 | Ref | Ref |  |
|  | **2 SDH** | 821/25 | 1.03 (0.62 – 1.69) | 861/118 | 1.26 (1.01 – 1.58) * | 1.23 (1.00 – 1.51) * |  |
|  | **3 SDH** | 823/34 | 1.68 (0.98 – 2.86) | 477/89 | 1.91 (1.49 – 2.45) *** | 1.88 (1.50 – 2.35) *** |  |
|  | **≥ 4 SDH** | 593/47 | 2.75 (1.56 – 4.83) ** | 375/82 | 2.75 (2.11 – 3.57) *** | 2.79 (2.20 – 3.53) *** |  |
| **e4** | **≤ 1 SDH** | 319/16 | 1.36 (0.73 – 2.52) | 762/132 | 1.73 (1.39 – 2.15) *** | 1.70 (1.39 – 2.08) *** |  |
|  | **2 SDH** | 401/22 | 1.63 (0.93 – 2.88) | 370/67 | 1.96 (1.49 – 2.59) *** | 1.91 (1.50 – 2.44) *** |  |
|  | **3 SDH** | 231/16 | 2.00 (1.08 – 3.72) * | 241/54 | 2.75 (2.03 – 3.71) *** | 2.58 (1.97 – 3.37) *** |  |
|  | **≥ 4 SDH** | 142/15 | 3.33 (1.76 – 6.30) *** | 159/33 | 2.57 (1.76 – 3.75) *** | 2.82 (2.05 – 3.88) *** |  |

HRS: the Health and Retirement Study; ELSA: the English Longitudinal Study on Aging; SDH: Social Determinants of Health; HR: Hazard ratio; CI: Confidence Interval; I^2^: Random effect; Ref: reference value.

All models were adjusted by age, sex, and race. The pooled models were additionally adjusted by cohort.

*: p < 0.05; **: p < 0.01; ***: p < 0.001

Social adversity measured as N of unfavorable SDH exposed: Less than upper secondary education, being in the 25^th^ lower family income range, 25^th^ lower score in neighborhood physical disorder, 25^th^ lower score in neighborhood social cohesion, having experienced poorer health service from a doctor or hospital, not having some private healthcare insurance, 25^th^ lower score in perceived social support, having experienced two or more different types of discrimination.

Dementia risk factors assessed: hypertension, diabetes, obesity, smoking, high-frequency drinking (drinking more than 5 days a week), physical inactivity (participating <1 time per week in moderate or vigorous physical activity), self-reported hearing problems, and depression (CES-D score >2 points).

Model properties: all the models' long-rank test p-value < 0.001. Fully adjusted model hazard proportionality assumption based on Schoenfeld residuals. The models meet the hazard proportionality assumption (p-value >0.05). All the variables meet the hazard proportionality assumption (p-value >0.05).

| **S7 Table. Descriptive statistics of ELSA and HRS sample included and excluded based on complete social determinants of health information available at baseline** | | | | | | |
| --- | --- | --- | --- | --- | --- | --- |
|  | **ELSA** | | **P value** | **HRS** | | **P value** |
|  | Included complete SDH  (n: 4,330) | Excluded incomplete SDH  (n: 1,201) |  | Included complete SDH  (n: 7,530) | Excluded incomplete SDH  (n: 11,386) |  |
| Age  Mean, SD (IQR) | 67.98, 8.66 (61 – 74) | 70.81, 10.63 (62 – 79) | 2.2e^-16^ | 65.00, 11.11 (56 – 73) | 62.66, 10.94 (54 – 70) | 2.2e^-16^ |
| Sex n (%) |  |  |  |  |  |  |
| Male | 2016 (46.6%) | 473 (39.4%) | 9.765e^-05^ | 3152 (41.9%) | 4802 (42.2%) | 0.6671 |
| Female | 2314 (53.4%) | 728 (60.6%) |  | 4378 (58.1%) | 6584 (57.8%) |  |
| Race/ethnicity n (%) |  |  |  |  |  |  |
| Non-Hispanic White | 4229 (99.9%) | 1201 (100%) | NA | 6010 (79.9%) | 8051 (70.9%) | 2.2e^-16^ |
| Non-Hispanic Black | 0 | 0 |  | 1082 (14.4%) | 2303 (20.3%) |  |
| Other (American  Indian, Alaskan  Native, Asian, and  Pacific Islander) | 1 (0.0%) | 0 |  | 434 (5.8%) | 997 (8.8%) |  |
| Hispanic | 0 | 0 |  | 686 (9.1%) | 1705 (15.0%) |  |
| Dementia risk factors n (%) |  |  |  |  |  |  |
| Diabetes | 413 (9.5%) | 146 (12.2%) | 0.007728 | 1365 (18.1%) | 2116 (18.6%) | 0.4275 |
| Hypertension | 1858 (42.9%) | 580 (48.3%) | 0.000885 | 3923 (52.1%) | 5707 (50.1%) | 0.00781 |
| Obesity | 1218 (31.0%) | 297 (30.7%) | 0.8593 | 2505 (33.7%) | 3808 (34.1%) | 0.6071 |
| High-frequency drinking | 772 (18.2%) | 107 (14.4%) | 0.01276 | 640 (8.5%) | 832 (7.3%) | 0.002927 |
| No or little participation in physical activity | 897 (20.7%) | 414 (34.5%) | 2.2e^-16^ | 1873 (24.9%) | 2966 (26.1%) | 0.0694 |
| Smoking | 463 (10.7%) | 143 (12.4%) | 0.106 | 1083 (14.5%) | 2068 (18.3%) | 7.888e^-16^ |
| Hearing problems | 184 (4.3%) | 93 (7.8%) | 8.776e^-07^ | 323 (4.3%) | 456 (4.0%) | 0.3143 |
| Depression | 825 (19.1%) | 288 (27.7%) | 8.597e^-09^ | 1561 (20.7%) | 2751 (24.6%) | 5.568e^-16^ |
| N of dementia risk factors n (%) |  |  |  |  |  |  |
| 0 | 921 (21.3%) | 233 (19.4%) | 2.454e^-08^ | 1292 (17.2%) | 1905 (16.7%) | 0.02004 |
| 1 | 1445 (33.4%) | 328 (27.3%) |  | 2234 (29.7%) | 3185 (28.0%) |  |
| 2 | 1062 (24.5%) | 290 (24.1%) |  | 1886 (25.0%) | 2884 (25.3%) |  |
| ≥3 | 902 (20.8%) | 350 (29.2%) |  | 2118 (28.2%) | 3412 (30.0%) |  |
| Social determinants of health |  |  |  |  |  |  |
| Education level n (%) |  |  |  |  |  |  |
| Less than upper secondary | 1336 (30.9%) | 343 (45.6%) | 2.291e^-14^ | 1254 (16.7%) | 2523 (22.2%) | 2.2e^-16^ |
| Upper secondary and vocational | 2217 (51.2%) | 308 (40.9%) |  | 4493 (59.7%) | 6594 (57.9%) |  |
| Tertiary | 777 (17.9%) | 102 (13.5%) |  | 1783 (23.7%) | 2267 (19.9%) |  |
| Family income quartile by cohort n (%) |  |  |  |  |  |  |
| <25^th^ quartile income | 979 (22.6%) | 359 (30.5%) | 6.324e^-14^ | 1568 (20.8%) | 2693 (23.7%) | 3.311e^-08^ |
| 25^th^ – 75^th^ quartile income | 2224 (51.4%) | 626 (53.2%) |  | 3731 (49.5%) | 5698 (50.0%) |  |
| >75^th^ quartile income | 1127 (26.0%) | 192 (16.3%) |  | 2231 (29.6%) | 2995 (26.3%) |  |
| Healthcare access n (%) |  |  |  |  |  |  |
| No private insurance | 3760 (86.8%) | 1075 (89.5%) | 0.2047 | 2107 (28.0%) | 3741 (33.1%) | 1.129e^-13^ |
| Healthcare discrimination | 186 (4.3%) | 23 (3.4%) | 0.2565 | 627 (8.3%) | 55 (9.9%) | 0.01347 |
| Neighborhood environment n (%) |  |  |  |  |  |  |
| Physical disorder |  |  |  |  |  |  |
| Low | 779 (18.0%) | 221 (24.1%) | 1.174e^-05^ | 1864 (25.0%) | 233 (32.7%) | 1.817e^-05^ |
| Moderate | 2116 (48.9%) | 436 (48.0%) |  | 3323 (44.5%) | 298 (41.9%) |  |
| High | 1435 (33.1%) | 252 (27.7%) |  | 2276 (30.5%) | 181 (18.1%) |  |
| Social cohesion difficulties |  |  |  |  |  |  |
| Low | 1319 (30.5%) | 295 (32.3%) | 0.4912 | 1893 (25.3%) | 244 (33.7%) | 1.16e^-06^ |
| Moderate | 2206 (50.9%) | 457 (50.1%) |  | 3365 (45.0%) | 311 (43.0%) |  |
| High | 805 (18.6%) | 160 (17.5%) |  | 2214 (29.6%) | 169 (23.3%) |  |
| Social and community context n (%) |  |  |  |  |  |  |
| Social support |  |  |  |  |  |  |
| Low | 1047 (24.2%) | 302 (28.3%) | 0.003525 | 1977 (26.3%) | 211 (33.7%) | 0.000188 |
| Moderate | 2335 (53.9%) | 518 (48.5%) |  | 3718 (49.4%) | 289 (46.1%) |  |
| High | 1047 (24.2%) | 302 (28.3%) |  | 1835 (24.4%) | 127 (20.3%) |  |
| N of types of discrimination |  |  |  |  |  |  |
| 0 | 2409 (55.6%) | 370 (54.7%) | 0.355607 | 3036 (40.3%) | 188 (33.8%) | 0.005136 |
| 1 | 1264 (29.2%) | 214 (31.6%) |  | 2737 (36.3%) | 235 (42.2%) |  |
| ≥2 | 657 (15.2%) | 93 (13.7%) |  | 1757 (23.3%) | 134 (24.0%) |  |
| APOE allele dosage |  |  |  |  |  |  |
| e2e2 | 21 (0.5%) | 6 (0.5%) | 0.2269 | 46 (0.6%) | 77 (0.7%) | 0.1098 |
| e2e3 | 561 (13.0%) | 138 (11.5%) |  | 895 (11.9%) | 1431 (12.6%) |  |
| e2e4 | 0 (0.0%) | 0 (0.0%) |  | 182 (2.4%) | 276 (2.4%) |  |
| e3e3 | 2574 (59.4%) | 707 (58.9%) |  | 4572 (60.7%) | 6747 (59.3%) |  |
| e3e4 | 985 (22.7%) | 291 (24.2%) |  | 1663 (22.1%) | 2610 (22.9%) |  |
| e4e4 | 189 (4.4%) | 59 (4.9%) |  | 172 (2.3%) | 245 (2.2%) |  |
| Overall APOE dosage |  |  |  |  |  |  |
| e2 (e2e2, e2e3) | 582 (13.4%) | 144 (12.0%) | 0.5284 | 941 (12.5%) | 1508 (13.2%) | 0.3692 |
| e3e3 | 2574 (59.4%) | 707 (58.9%) |  | 4572 (60.7%) | 6747 (59.3%) |  |
| e4 (e2e4, e3e4, e4e4) | 1174 (27.1%) | 350 (29.1%) |  | 2017 (26.8%) | 3131 (27.5%) |  |
| Any functional limitations in ADL n (%) | 680 (15.7%) | 345 (28.7%) | 2.2e-16 | 14.7 (14.7%) | 1800 (15.8%) | 0.000511 |
| Dementia n (%) | 124 (2.9%) | 141 (21.2%) | 2.2e-16 | 164 (2.2%) | 308 (3.0%) | 0.03636 |

HRS: the Health and Retirement Study; ELSA: the English Longitudinal Study on Aging; SDH: Social Determinants of Health; IQR: interquartile range; NA: not applicable.

*Excluding people 55 years or less.

<25^th^: less than 25^th^ quartile by cohort; 25^th^ – 75^th^: between 25^th^ and 75^th^ quartile by cohort; >75^th^: more than 75^th^ quartile by cohort.

P-value for chi-square test (X^2^) for nominal and ordinal variables and ANOVA test for continuous variables comparison.

| **S8.a Table. Analysis of social adversity on the risk of dementia according to APOE allele risk profile weighting by the inverse probability of being selected in the study.** | | | | | | | |
| --- | --- | --- | --- | --- | --- | --- | --- |
|  |  | **ELSA** | | **HRS** | | **Fully adjusted Pooled analysis** | **I^2^** |
| **APOE dosage** | **N of SDH** | **N at risk / N event** | **HR (95%CI), p-value** | **N at risk / N event** | **HR (95%CI), p-value** | **HR (95%CI), p-value** |  |
| **e2** | **0 - 1 SDH** | 163/0 | Ref | 395/36 | Ref | Ref |  |
|  | **2 SDH** | 185/8 | 3.42 (1.64 – 7.11) *** | 154/16 | 1.28 (0.68 – 2.42) | 1.65 (1.00 – 2.71) * | 0.0% |
|  | **3 SDH** | 111/8 | 6.40 (2.99 – 13.71) *** | 77/12 | 2.15 (1.03 – 4.50) * | 2.92 (1.72 – 4.95) *** |  |
|  | **≥ 4 SDH** | 79/3 | 4.07 (1.17 – 14.15) *** | 69/16 | 4.42 (2.15 – 9.12) *** | 4.42 (2.56 – 7.64) *** |  |
| **e3e3** | **0 - 1 SDH** | 693/27 | Ref | 1812/215 | Ref | Ref |  |
|  | **2 SDH** | 859/36 | 1.05 (0.64 – 1.72) | 844/112 | 1.22 (0.97 – 1.54) | 1.21 (0.99 – 1.49) | 0.0% |
|  | **3 SDH** | 516/28 | 1.73 (1.02 – 2.93) * | 467/88 | 2.05 (1.59 – 2.65) *** | 2.01 (1.62 – 2.50) *** |  |
|  | **≥ 4 SDH** | 297/22 | 3.17 (1.72 – 5.32) *** | 372/82 | 3.59 (2.70 – 4.77) *** | 3.55 (2.86 – 4.40) *** |  |
| **e4** | **0 - 1 SDH** | 315/16 | Ref | 752/130 | Ref | Ref |  |
|  | **2 SDH** | 394/22 | 1.36 (0.73 – 2.54) | 362/65 | 1.08 (0.80 – 1.47) | 1.13 (0.87 – 1.46) | 0.0% |
|  | **3 SDH** | 223/15 | 1.51 (0.77 – 2.96) | 237/54 | 2.01 (1.43 – 2.84) *** | 1.88 (1.43 – 2.47) *** |  |
|  | **≥ 4 SDH** | 140/14 | 2.97 (1.44 – 6.12) ** | 155/31 | 2.10 (1.31 – 3.39) ** | 2.25 (1.64 – 3.07) *** |  |
| **P for interaction** | |  | 0.21807 |  | 0.0211 | 0.00075 |  |

HRS: the Health and Retirement Study; ELSA: the English Longitudinal Study on Aging; SDH: Social Determinants of Health; HR: Hazard ratio; CI: Confidence Interval; I^2^: Random effect; Ref: reference value.

All models were adjusted by age, sex, and race. The pooled models were additionally adjusted by cohort.

*: p < 0.05; **: p < 0.01; ***: p < 0.001

Social adversity measured as N of unfavorable SDH exposed: Less than upper secondary education, being in the 25^th^ lower family income range, 25^th^ lower score in neighborhood physical disorder, 25^th^ lower score in neighborhood social cohesion, having experienced poorer health service from a doctor or hospital, not having some private healthcare insurance, 25^th^ lower score in perceived social support, having experienced two or more different types of discrimination.

Model properties: all the models' long-rank test p-value < 0.001. Fully adjusted model hazard proportionality assumption based on Schoenfeld residuals. The models meet the hazard proportionality assumption (p-value >0.05). All the variables meet the hazard proportionality assumption (p-value >0.05).

| **S8.b Table. Analysis of social adversity on the risk of dementia according to APOE allele risk profile weighting by the inverse probability of being selected in the study.** | | | | | | | |
| --- | --- | --- | --- | --- | --- | --- | --- |
|  |  | **ELSA** | | **HRS** | | **Fully adjusted pooled analysis** | **I^2^** |
| **APOE dosage** | **N of SDH** | **N total / N events** | **HR (95%CI), p-value** | **N total / N events** | **HR (95%CI), p-value** | **HR (95%CI), p-value** | 0.0% |
| **e2** | **0 - 1 SDH** | 163/0 | 0.05 (0.00 – 0.08) *** | 395/36 | 0.75 (0.52 – 1.08) | 0.65 (0.47 – 0.91) * |  |
|  | **2 SDH** | 185/8 | 1.18 (0.54 – 2.61) | 154/16 | 1.00 (0.59 – 1.69) | 1.10 (0.75 – 1.42) |  |
|  | **3 SDH** | 111/8 | 2.11 (0.90 – 4.94) | 77/12 | 1.70 (0.89 – 3.26) | 1.93 (1.26 – 2.95) ** |  |
|  | **≥ 4 SDH** | 79/3 | 1.40 (0.41 – 4.76) | 69/16 | 3.73 (2.11 – 6.59) *** | 3.17 (2.08 – 4.83) *** |  |
| **e3e3** | **0 - 1 SDH** | 693/27 | Ref | 1812/215 | Ref | Ref |  |
|  | **2 SDH** | 859/36 | 1.08 (0.66 – 1.76) | 844/112 | 1.23 (0.98 – 1.55) | 1.23 (1.01 – 1.50) * |  |
|  | **3 SDH** | 516/28 | 1.79 (1.06 – 3.03) | 467/88 | 2.07 (1.60 – 2.67) *** | 2.04 (1.65 – 2.53) *** |  |
|  | **≥ 4 SDH** | 297/22 | 3.20 (1.82 – 5.64) | 372/82 | 3.65 (2.73 – 4.86) *** | 3.62 (2.94 – 4.46) *** |  |
| **e4** | **0 - 1 SDH** | 315/16 | 1.32 (0.72 – 2.41) | 752/130 | 1.65 (1.33 – 2.06) *** | 1.63 (1.34 – 1.99) *** |  |
|  | **2 SDH** | 394/22 | 1.77 (1.03 – 3.06) | 362/65 | 1.74 (1.30 – 2.32) *** | 1.78 (1.40 – 2.26) *** |  |
|  | **3 SDH** | 223/15 | 1.94 (1.06 – 3.54) | 237/54 | 3.18 (2.26 – 4.47) *** | 2.92 (2.27 – 3.75) *** |  |
|  | **≥ 4 SDH** | 140/14 | 3.75 (1.93 – 7.28) | 155/31 | 3.21 (1.98 – 5.20) *** | 3.38 (2.54 – 4.50) *** |  |

HRS: the Health and Retirement Study; ELSA: the English Longitudinal Study on Aging; SDH: Social Determinants of Health; HR: Hazard ratio; CI: Confidence Interval; I^2^: Random effect; Ref: reference value.

All models were adjusted by age, sex, and race. The pooled models were additionally adjusted by cohort.

*: p < 0.05; **: p < 0.01; ***: p < 0.001

Social adversity measured as N of unfavorable SDH exposed: Less than upper secondary education, being in the 25^th^ lower family income range, 25^th^ lower score in neighborhood physical disorder, 25^th^ lower score in neighborhood social cohesion, having experienced poorer health service from a doctor or hospital, not having some private healthcare insurance, 25^th^ lower score in perceived social support, having experienced two or more different types of discrimination.

Model properties: all the models' long-rank test p-value < 0.001. Fully adjusted model hazard proportionality assumption based on Schoenfeld residuals. The models meet the hazard proportionality assumption (p-value >0.05). All the variables meet the hazard proportionality assumption (p-value >0.05).

| **S9.a Table. Analysis of social disadvantage on the risk of dementia according to APOE allele risk profile by sex.** | | | | | | | | | | | | |
| --- | --- | --- | --- | --- | --- | --- | --- | --- | --- | --- | --- | --- |
|  |  | **Male** | | | | **Male fully adjusted pooled analysis** | **Female** | | | | **Female fully adjusted pooled analysis** | **I^2^** |
|  |  | **ELSA** | | **HRS** | |  | **ELSA** | | **HRS** | |  |  |
| **APOE dosage** | **N of SDH** | **N total / N events** | **HR (95%CI), p-value** | **N total / N events** | **HR (95%CI), p-value** | **HR (95%CI), p-value** | **N total / N events** | **HR (95%CI), p-value** | **N total / N events** | **HR (95%CI), p-value** | **HR (95%CI), p-value** |  |
| **e2** | **0 SDH** | 25/0 | NA | 156/16 | Ref | Ref | 15/0 | NA | 247/21 | Ref | Ref |  |
|  | **1 SDH** | 93/3 | Ref | 61/6 | 1.19 (0.46 – 3.06) | 1.01 (0.44 – 2.35) | 82/1 | Ref | 97/12 | 1.57 (0.76 – 3.25) | 1.56 (0.77 – 3.18) | M: 0.0%  F: 0.0% |
|  | **2 SDH** | 91/5 | 1.60 (0.38 - .77) | 31/4 | 1.83 (0.60 – 5.56) | 1.51 (0.65 – 3.52) | 91/4 | 3.72 (0.41 – 33.69) | 46/8 | 2.87 (1.23 – 6.67) * | 3.48 (1.63 – 7.44) ** |  |
|  | **≥ 3SDH** | 60/6 | 3.87 (0.97 – 15.48) | 31/4 | 1.64 (0.53 – 5.08) | 2.40 (1.08 – 5.37) * | 85/3 | 2.87 (0.28 – 29.03) | 38/12 | 9.81 (4.43 – 21.72) *** | 8.38 (3.87 – 18.17) *** |  |
| **e3e3** | **0 SDH** | 96/4 | Ref | 816/82 | Ref | Ref | 2/4 | Ref | 1029/140 | Ref | Ref |  |
|  | **1 SDH** | 418/14 | 0.90 (0.30 – 2.76) | 374/45 | 1.47 (1.02 – 2.12) * | 1.42 (1.01 – 2.00) * | 77/4 | 0.51 (0.16 – 1.62) | 487/73 | 1.56 (0.77 – 3.18) | 1.20 (0.91 – 1.58) | M: 0.0%  F: 0.0% |
|  | **2 SDH** | 347/13 | 0.93 (0.30 – 2.87) | 214/39 | 2.29 (1.55 – 3.37) *** | 1.94 (1.34 – 2.79) *** | 403/11 | 0.62 (0.21 – 1.82) | 263/50 | 3.48 (1.63 – 7.44) ** | 1.81 (1.33 – 2.47) *** |  |
|  | **≥ 3SDH** | 257/22 | 2.94 (1.01 – 8.66) * | 167/29 | 2.85 (1.84 – 4.42) *** | 3.29 (2.27 – 4.78) *** | 476/21 | 1.65 (0.57 – 4.74) | 208/53 | 8.38 (3.87 – 18.17) *** | 3.19 (2.35 – 4.34) *** |  |
| **e4** | **0 SDH** | 37/1 | Ref | 324/52 | Ref | Ref | 34/1 | Ref | 438/80 | Ref | Ref |  |
|  | **1 SDH** | 190/9 | 1.00 (0.12 – 7.95) | 163/22 | 0.97 (0.59 – 1.59) | 0.95 (0.60 – 1.50) | 169/6 | 0.67 (0.07 – 5.73) | 207/45 | 1.28 (0.88 – 1.86) | 1.24 (0.86 – 1.78) | M: 0.0%  F: 0.0% |
|  | **2 SDH** | 179/16 | 2.13 (0.28 – 16.16) | 90/20 | 1.82 (1.08 – 3.08) * | 1.84 (1.16 – 2.93) * | 227/12 | 1.11 (0.14 – 8.66) | 151/34 | 1.83 (1.22 – 2.75) ** | 1.75 (1.19 – 2.58) ** |  |
|  | **≥ 3SDH** | 111/11 | 2.53 (0.32 – 19.89) | 67/13 | 2.14 (1.13 – 4.04) * | 2.20 (1.29 – 3.75) ** | 146/13 | 2.05 (0.26 – 16.08) | 92/20 | 1.64 (0.97 – 2.79) | 2.01 (1.28 – 3.16) ** |  |

HRS: the Health and Retirement Study; ELSA: the English Longitudinal Study on Aging; SDH: Social Determinants of Health; HR: Hazard ratio; CI: Confidence Interval; I2: Random effect; Ref: reference value.

All models were adjusted by age, sex, and race. The pooled models were additionally adjusted by cohort.

HRS: the Health and Retirement Study; ELSA: the English Longitudinal Study on Aging; SDH: Social Determinants of Health; HR: Hazard ratio; CI: Confidence Interval; I^2^: Random effect; Ref: reference value.

All models were adjusted by age, sex, and race. The pooled models were additionally adjusted by cohort.

*: p < 0.05; **: p < 0.01; ***: p < 0.001

Social adversity measured as N of unfavorable SDH exposed: Less than upper secondary education, being in the 25^th^ lower family income range, 25^th^ lower score in neighborhood physical disorder, 25^th^ lower score in neighborhood social cohesion, having experienced poorer health service from a doctor or hospital, not having some private healthcare insurance, 25^th^ lower score in perceived social support, having experienced two or more different types of discrimination.

Model properties: all the models' long-rank test p-value < 0.001. Fully adjusted model hazard proportionality assumption based on Schoenfeld residuals. The models meet the hazard proportionality assumption (p-value >0.05). All the variables meet the hazard proportionality assumption (p-value >0.05).

| **S9.b Table. Analysis of social disadvantage on the risk of dementia according to APOE allele risk profile by sex.** | | | | | | | | | | | | |
| --- | --- | --- | --- | --- | --- | --- | --- | --- | --- | --- | --- | --- |
|  |  | **Male** | | | | **Male fully adjusted pooled analysis** | **Female** | | | | **Female fully adjusted pooled analysis** | **I^2^** |
|  |  | **ELSA** | | **HRS** | |  | **ELSA** | | **HRS** | |  |  |
| **APOE dosage** | **N of SDH** | **N total / N events** | **HR (95%CI), p-value** | **N total / N events** | **HR (95%CI), p-value** | **HR (95%CI), p-value** | **N total / N events** | **HR (95%CI), p-value** | **N total / N events** | **HR (95%CI), p-value** | **HR (95%CI), p-value** | M: 5.5%  F: 10.2% |
| **e2** | **0 SDH** | 25/0 | NA | 156/16 | 0.97 (0.57 – 1.66) | 0.93 (0.54 – 1.58) | 15/0 | 0.0 (0.0 – 0.0) | 247/21 | 0.61 (0.38 – 0.96) * | 0.59 (0.38 – 0.94) * |  |
|  | **1 SDH** | 93/3 | 0.74 (0.17 – 3.32) | 61/6 | 1.15 (0.50 – 2.63) | 1.07 (0.54 – 2.13) | 82/1 | 0.18 (0.02 – 1.66) | 97/12 | 0.93 (0.51 – 1.68) | 0.84 (0.48 – 1.49) |  |
|  | **2 SDH** | 91/5 | 1.24 (0.33 – 4.62) | 31/4 | 1.50 (0.55 – 4.10) | 1.58 (0.79 – 3.17) | 91/4 | 0.70 (0.18 – 2.82) | 46/8 | 1.70 (0.83 – 3.47) | 1.71 (0.94 – 3.11) |  |
|  | **≥ 3SDH** | 60/6 | 2.93 (0.82 – 10.41) | 31/4 | 1.98 (0.72 – 5.44) | 2.81 (1.44 – 5.46) ** | 85/3 | 0.55 (0.12 – 2.45) | 38/12 | 5.64 (3.10 – 10.26) *** | 3.69 (2.13 – 6.37) *** |  |
| **e3e3** | **0 SDH** | 96/4 | Ref | 816/82 | Ref | Ref | 2/4 | Ref | 1029/140 | Ref | Ref |  |
|  | **1 SDH** | 418/14 | 0.88 (0.30 – 2.79) | 374/45 | 1.49 (1.03 – 2.15) | 1.40 (1.00 – 1.96) | 77/4 | 0.51 (0.16 – 1.60) | 487/73 | 1.23 (0.93 – 1.64) | 1.22 (0.93 – 1.60) |  |
|  | **2 SDH** | 347/13 | 0.90 (0.30 – 2.79) | 214/39 | 2.31 (1.57 – 3.39) | 1.86 (1.31 – 2.66) *** | 403/11 | 0.62 (0.21 – 1.81) | 263/50 | 1.98 (1.43 – 2.75) *** | 1.88 (1.40 – 2.53) *** |  |
|  | **≥ 3SDH** | 257/22 | 2.86 (0.98 – 8.35) | 167/29 | 2.90 (1.89 – 4.47) | 3.22 (2.25 – 4.61) *** | 476/21 | 1.62 (0.56 – 4.65) | 208/53 | 3.13 (2.25 – 4.34) *** | 3.32 (2.48 – 4.46) *** |  |
| **e4** | **0 SDH** | 37/1 | 1.12 (0.12 – 10.06) | 324/52 | 1.78 (1.26 – 2.53) | 1.76 (1.23 – 2.48) ** | 34/1 | 1.02 (0.11 – 9.21) | 438/80 | 1.67 (1.26 – 2.19) *** | 1.66 (1.26 – 2.18) *** |  |
|  | **1 SDH** | 190/9 | 1.20 (0.37 – 3.91) | 163/22 | 1.69 (1.05 – 2.71) | 1.64 (1.08 – 2.49) * | 169/6 | 0.59 (0.17 – 2.12) | 207/45 | 2.07 (1.47 – 2.90) *** | 1.97 (1.42 – 2.73) *** |  |
|  | **2 SDH** | 179/16 | 2.50 (0.83 – 7.49) | 90/20 | 3.05 (1.86 – 5.02) | 3.09 (2.06 – 4.64) *** | 227/12 | 1.02 (0.33 – 3.18) | 151/34 | 2.95 (2.02 – 4.31) *** | 2.78 (1.97 – 3.92) *** |  |
|  | **≥ 3SDH** | 111/11 | 2.79 (0.88 – 8.80) | 67/13 | 3.27 (1.80 – 5.95) | 3.47 (2.17 – 5.53) *** | 22/1 | 1.69 (0.55 – 5.19) | 34/6 | 2.59 (2.02 – 4.31) *** | 3.07 (2.06 – 4.56) *** |  |
| **P for interaction** | |  | 0.852 |  | 0.110 | 0.150 |  | 0.912 |  | 0.003 | 0.004 |  |

HRS: the Health and Retirement Study; ELSA: the English Longitudinal Study on Aging; SDH: Social Determinants of Health; HR: Hazard ratio; CI: Confidence Interval; I^2^: Random effect; Ref: reference value.

All models were adjusted by age, sex, and race. The pooled models were additionally adjusted by cohort.

*: p < 0.05; **: p < 0.01; ***: p < 0.001

Social adversity measured as N of unfavorable SDH exposed: Less than upper secondary education, being in the 25^th^ lower family income range, 25^th^ lower score in neighborhood physical disorder, 25^th^ lower score in neighborhood social cohesion, having experienced poorer health service from a doctor or hospital, not having some private healthcare insurance, 25^th^ lower score in perceived social support, having experienced two or more different types of discrimination.

Model properties: all the models' long-rank test p-value < 0.001. Fully adjusted model hazard proportionality assumption based on Schoenfeld residuals. The models meet the hazard proportionality assumption (p-value >0.05). All the variables meet the hazard proportionality assumption (p-value >0.05).

| **S10.a Table. Analysis of social disadvantage on the risk of dementia according to APOE allele risk profile by race.** | | | | | | | | | |
| --- | --- | --- | --- | --- | --- | --- | --- | --- | --- |
|  |  | **Black people** | | **White Caucasian people** | | | | **White people adjusted pooled analysis** | **I^2^** |
|  |  | **HRS n: 668** | | **ELSA n: 4,052** | | **HRS n: 4,781** | |  |  |
| **APOE dosage** | **N of SDH** | **N total / N events** | **HR (95%CI), p-value** | **N total / N events** | **HR (95%CI), p-value** | **N total / N events** | **HR (95%CI), p-value** | **HR (95%CI), p-value** |  |
| **e2** | **0 - 1 SDH** | 38/7 | Ref | 168/2 | Ref | 355/28 | Ref | Ref |  |
|  | **2 SDH** | 23/2 | 0.34 (0.07 – 1.74) | 186/8 | 4.08 (0.86 – 19.34) | 130/15 | 1.85 (0.98 – 3.47) | 1.79 (0.99 – 3.25) | W: 0.0% |
|  | **3 SDH** | 17/3 | 1.19 (0.30 – 4.74) | 113/8 | 7.48 (1.58 – 35.34)  ** | 59/9 | 2.80 (1.31 – 5.98) ** | 3.18 (1.68 – 6.04) *** |  |
|  | **≥ 4 SDH** | 26/6 | 2.10 (0.69 – 6.34) | 82/4 | 6.37 (1.14 – 35.49)  ** | 39/9 | 7.06 (3.19 – 15.61) *** | 6.02 (3.07 – 11.83) *** |  |
| **e3e3** | **0 - 1 SDH** | 102/13 | Ref | 704/28 | Ref | 1697/205 | Ref | Ref |  |
|  | **2 SDH** | 103/23 | 1.66 (0.84 – 3.29) | 878/36 | 1.06 (0.64 – 1.75) | 712/89 | 1.29 (1.01 – 1.65) * | 1.25 (0.99 – 1.57) | W: 0.0% |
|  | **3 SDH** | 78/26 | 3.09 (1.58 – 6.04) *** | 527/28 | 1.79 (1.05 – 3.05)  * | 364/61 | 2.00 (1.50 – 2.66) *** | 1.70 (1.31 – 2.21) *** |  |
|  | **≥ 4 SDH** | 111/33 | 3.73 (1.96 – 711) *** | 301/23 | 3.23 (1.85 – 5.66)  *** | 224/39 | 2.65 (1.88 – 3.74) *** | 3.08 (2.35 – 4.05) *** |  |
| **e4** | **0 - 1 SDH** | 71/10 | Ref | 319/16 | Ref | 669/120 | Ref | Ref |  |
|  | **2 SDH** | 69/16 | 1.69 (0.76 – 3.76) | 401/22 | 1.32 (0.69 – 2.52) | 289/51 | 1.16 (0.83 – 1.61) | 1.09 (0.80 – 1.48) | W: 0.0% |
|  | **3 SDH** | 66/13 | 2.15 (0.93 – 4.94) | 231/16 | 1.58 (0.79 – 3.17) | 164/37 | 1.73 (1.19 – 2.51) ** | 1.69 (1.21 – 2.35) ** |  |
|  | **≥ 4 SDH** | 62/18 | 2.59 (1.19 – 5.64) * | 142/15 | 3.14 (1.55 – 6.40)  *** | 79/12 | 1.48 (0.81 – 2.68) | 2.00 (1.33 – 3.01) *** |  |

HRS: the Health and Retirement Study; ELSA: the English Longitudinal Study on Aging; SDH: Social Determinants of Health; HR: Hazard ratio; CI: Confidence Interval; I^2^: Random effect; Ref: reference value.

All models were adjusted by age, sex, and race. The pooled models were additionally adjusted by cohort.

*: p < 0.05; **: p < 0.01; ***: p < 0.001

All models were adjusted by age, sex, and race. The pooled models were additionally adjusted by cohort.

Social adversity measured as N of unfavorable SDH exposed: Less than upper secondary education, being in the 25^th^ lower family income range, 25^th^ lower score in neighborhood physical disorder, 25^th^ lower score in neighborhood social cohesion, having experienced poorer health service from a doctor or hospital, not having some private healthcare insurance, 25^th^ lower score in perceived social support, having experienced two or more different types of discrimination.

Model properties: all the models' long-rank test p-value < 0.001. Fully adjusted model hazard proportionality assumption based on Schoenfeld residuals. The models meet the hazard proportionality assumption (p-value >0.05). All the variables meet the hazard proportionality assumption (p-value >0.05).

| **S10.b Table. Analysis of social disadvantage on the risk of dementia according to APOE allele risk profile by race.** | | | | | | | | | |
| --- | --- | --- | --- | --- | --- | --- | --- | --- | --- |
|  |  | **Black people** | | **White people** | | | | **White people adjusted pooled analysis** | **I^2^** |
|  |  | **HRS n: 766** | | **ELSA n: 4,052** | | **HRS n: 4,781** | |  |  |
| **APOE dosage** | **N of SDH** | **N total / N events** | **HR (95%CI), p-value** | **N total / N events** | **HR (95%CI), p-value** | **N total / N events** | **HR (95%CI), p-value** | **HR (95%CI), p-value** | 0.5% |
| **e2** | **0 - 1 SDH** | 38/7 | 1.40 (0.56 – 3.52) | 168/2 | 0.28 (0.07 – 1.18) | 355/28 | 0.61 (0.41 – 0.90) * | 0.59 (0.40 – 0.87) ** |  |
|  | **2 SDH** | 23/2 | 0.48 (0.43 – 2.11) | 186/8 | 1.16 (0.53 – 2.56) | 130/15 | 1.14 (0.67 – 1.92) | 1.01 (0.63 – 1.62) |  |
|  | **3 SDH** | 17/3 | 1.77 (0.50 – 6.21) | 113/8 | 2.01 (0.91 – 4.43) | 59/9 | 1.63 (0.83 – 3.17) | 1.65 (1.01 – 2.68) * |  |
|  | **≥ 4 SDH** | 26/6 | 3.45 (1.31 – 9.12) * | 82/4 | 1.77 (0.62 – 5.07) | 39/9 | 4.16 (2.13 – 8.14) *** | 3.13 (1.91 – 5.13) *** |  |
| **e3e3** | **0 - 1 SDH** | 102/13 | Ref | 704/28 | Ref | 1697/205 | Ref | Ref |  |
|  | **2 SDH** | 103/23 | 1.68 (0.85 – 3.32) | 878/36 | 1.08 (0.65 – 1.78) | 712/89 | 1.28 (1.00 – 1.64) | 1.26 (1.00 – 1.58) |  |
|  | **3 SDH** | 78/26 | 3.13 (1.61 – 6.11) *** | 527/28 | 1.85 (1.09 – 3.15)  * | 364/61 | 1.98 (1.49 – 2.64) *** | 1.73 (1.35 – 2.23) *** |  |
|  | **≥ 4 SDH** | 111/33 | 3.78 (1.98 – 7.19) *** | 301/23 | 3.26 (1.87 – 5.71)  *** | 224/39 | 2.62 (1.86 – 3.69) *** | 3.15 (2.42 – 4.10) *** |  |
| **e4** | **0 - 1 SDH** | 71/10 | 1.16 (0.51 – 2.65) | 319/16 | 1.36 (0.73 – 2.52) | 669/120 | 1.80 (1.44 – 2.26) *** | 1.78 (1.43 – 2.23) *** |  |
|  | **2 SDH** | 69/16 | 1.88 (0.90 – 3.91) | 401/22 | 1.77 (1.01 – 3.11)  * | 289/51 | 2.09 (1.53 – 2.84) *** | 1.92 (1.45 – 2.54) *** |  |
|  | **3 SDH** | 66/13 | 2.40 (1.11 – 5.21) * | 231/16 | 2.10 (1.13 – 3.90)  ** | 164/37 | 3.10 (2.18 – 4.40) *** | 2.96 (2.22 – 3.95) *** |  |
|  | **≥ 4 SDH** | 62/18 | 2.97 (1.45 – 6.07) ** | 142/15 | 4.09 (2.17 – 7.71)  *** | 79/12 | 2.65 (1.48 – 4.76) *** | 3.39 (2.35 – 4.90) *** |  |
| **P for interaction** | |  | 0.186 |  | 0.740 |  | 0.006 | 0.005 |  |

HRS: the Health and Retirement Study; ELSA: the English Longitudinal Study on Aging; SDH: Social Determinants of Health; HR: Hazard ratio; CI: Confidence Interval; I^2^: Random effect; Ref: reference value.

*: p < 0.05; **: p < 0.01; ***: p < 0.001

All models were adjusted by age, sex, and race. The pooled models were additionally adjusted by cohort.

Social adversity measured as N of unfavorable SDH exposed: Less than upper secondary education, being in the 25^th^ lower family income range, 25^th^ lower score in neighborhood physical disorder, 25^th^ lower score in neighborhood social cohesion, having experienced poorer health service from a doctor or hospital, not having some private healthcare insurance, 25^th^ lower score in perceived social support, having experienced two or more different types of discrimination.

Model properties: all the models' long-rank test p-value < 0.001. Fully adjusted model hazard proportionality assumption based on Schoenfeld residuals. The models meet the hazard proportionality assumption (p-value >0.05). All the variables meet the hazard proportionality assumption (p-value >0.05).

| **S11.a Table. Analysis of individual social determinants on the risk of dementia according to APOE allele risk profile by race.** | | | | | | | |
| --- | --- | --- | --- | --- | --- | --- | --- |
|  |  | **Fully adjusted Pooled analysis for education level** | |  | **Fully adjusted Pooled analysis for income level** | | **I^2^** |
| **APOE dosage** | **Education level** | **N at risk / N event** | **HR (95%CI), p-value** | **Family income** | **N at risk / N event** | **HR (95%CI), p-value** |  |
| **e2** | Tertiary education | 259/1 | Ref | High (>75th quartile) | 314/3 | Ref |  |
|  | Upper secondary and vocational | 668/38 | 14.72 (2.02 – 107.47) ** | Intermediate (25th to 75th quartile) | 637/38 | 5.22 (1.60 – 17.01) ** | 0.0% |
|  | Less than upper secondary school | 282/34 | 34.56 (1.45 – 33.70) *** | Low (<25th quartile) | 258/32 | 9.10 (2.70 – 30.60) *** |  |
| **e3e3** | Tertiary education | 1210/56 | Ref | High (>75th quartile) | 1419/52 | Ref |  |
|  | Upper secondary and vocational | 3180/226 | 1.56 (1.16 – 2.10) ** | Intermediate (25th to 75th quartile) | 3006/221 | 1.50 (1.11 – 2.04) ** | 0.0% |
|  | Less than upper secondary school | 1183/154 | 3.25 (2.37 – 4.45) *** | Low (<25th quartile) | 1148/163 | 2.76 (1.99 – 3.84) *** |  |
| **e4** | Tertiary education | 588/46 | Ref | High (>75th quartile) | 632/34 | Ref |  |
|  | Upper secondary and vocational | 1380/130 | 1.21 (0.86 – 1.70) | Intermediate (25th to 75th quartile) | 1332/148 | 1.54 (1.05 – 2.24) * | 0.0% |
|  | Less than upper secondary school | 502/79 | 2.21 (1.50 – 3.24) *** | Low (<25th quartile) | 506/73 | 2.01 (1.31 – 3.08) ** |  |
| P for interaction | |  | <0.001 |  |  | 0.005 |  |

HRS: the Health and Retirement Study; ELSA: the English Longitudinal Study on Aging; SDH: Social Determinants of Health; HR: Hazard ratio; CI: Confidence Interval; I^2^: Random effect; Ref: reference value.

All models were adjusted by age, sex, race, and cohort.

*: p < 0.05; **: p < 0.01; ***: p < 0.001

| **S11.b Table. Analysis of individual social determinants on the risk of dementia according to APOE allele risk profile by race.** | | | | | | | |
| --- | --- | --- | --- | --- | --- | --- | --- |
|  |  | **Fully adjusted Pooled analysis for healthcare access and quality** | |  | **Fully adjusted Pooled analysis for perceived social support** | | **I^2^** |
| **APOE dosage** | **Healthcare access and quality** | **N at risk / N event** | **HR (95%CI), p-value** | **Perceived social support** | **N at risk / N event** | **HR (95%CI), p-value** |  |
| **e2** | Adequate | 537/29 | Ref | High (>75th quartile) | 331/21 | Ref |  |
|  | Intermediate | 639/42 | 1.59 (0.92 – 2.76) | Intermediate (25th to 75th quartile) | 632/26 | 0.71 (0.40 – 1.29) | 0.0% |
|  | Low | 33/2 | 1.40 (0.32 – 6.09) | Low (<25th quartile) | 246/26 | 2.45 (1.35 – 4.42) ** |  |
| **e3e3** | Adequate | 2488/184 | Ref | High (>75th quartile) | 1482/128 | Ref |  |
|  | Intermediate | 2908/222 | 1.59 (1.28 – 1.98) *** | Intermediate (25th to 75th quartile) | 2901/205 | 1.03 (0.82 – 1.29) | 0.0% |
|  | Low | 177/30 | 3.47 (2.32 – 5.17) *** | Low (<25th quartile) | 1190/103 | 2.45 (1.35 – 4.42) *** |  |
| **e4** | Adequate | 1075/115 | Ref | High (>75th quartile) | 587/75 | Ref |  |
|  | Intermediate | 1306/129 | 1.49 (1.11 – 2.00) ** | Intermediate (25th to 75th quartile) | 1308/120 | 0.97 (0.72 – 1.30) | 0.0% |
|  | Low | 71/11 | 2.45 (1.30 – 4.59) ** | Low (<25th quartile) | 575/60 | 1.34 (0.95 – 1.90) |  |
| **P for interaction** | |  | <0.001 |  |  | 0.012 |  |

HRS: the Health and Retirement Study; ELSA: the English Longitudinal Study on Aging; SDH: Social Determinants of Health; HR: Hazard ratio; CI: Confidence Interval; I^2^: Random effect; Ref: reference value.

All models were adjusted by age, sex, race, and cohort.

*: p < 0.05; **: p < 0.01; ***: p < 0.001.

| **S11.c Table. Analysis of individual social determinants on the risk of dementia according to APOE allele risk profile by race.** | | | | | | | |
| --- | --- | --- | --- | --- | --- | --- | --- |
|  |  | **Fully adjusted Pooled analysis for physical environment** | |  | **Fully adjusted Pooled analysis for social cohesion** | | **I^2^** |
| **APOE dosage** | **Neighborhood physical disorder** | **N at risk / N event** | **HR (95%CI), p-value** | **Neighborhood social cohesion** | **N at risk / N event** | **HR (95%CI), p-value** |  |
| **e2** | Low (<25th quartile) | 422/16 | Ref | High (>75th quartile) | 399/25 | Ref |  |
|  | Intermediate (25th to 75th quartile) | 562/32 | 1.62 (0.90 – 3.00) | Intermediate (25th to 75th quartile) | 564/31 | 0.97 (0.57 – 1.66) | 0.0% |
|  | High (>75th quartile) | 225/25 | 3.34 (1.76 – 6.33) *** | Low (<25th quartile) | 246/17 | 1.51 (0.80 – 2.82) |  |
| **e3e3** | Low (<25th quartile) | 1846/127 | Ref | High (>75th quartile) | 1746/145 | Ref |  |
|  | Intermediate (25th to 75th quartile) | 2627/182 | 1.01 (0.81 – 1.27) | Intermediate (25th to 75th quartile) | 2715/187 | 0.97 (0.78 – 1.20) | 0.0% |
|  | High (>75th quartile) | 1100/127 | 1.71 (1.33 – 2.20) *** | Low (<25th quartile) | 1112/104 | 1.52 (1.17 – 1.96) ** |  |
| **e4** | Low (<25th quartile) | 775/69 | Ref | High (>75th quartile) | 749/83 | Ref |  |
|  | Intermediate (25th to 75th quartile) | 1172/121 | 1.18 (0.88 – 1.59) | Intermediate (25th to 75th quartile) | 1182/115 | 1.19 (0.89 – 1.59) | 0.0% |
|  | High (>75th quartile) | 523/65 | 1.24 (0.88 – 1.76) | Low (<25th quartile) | 539/57 | 1.17 (0.83 – 1.64) |  |
| **P for interaction** | |  | 0.533 |  |  | 0.934 |  |

HRS: the Health and Retirement Study; ELSA: the English Longitudinal Study on Aging; SDH: Social Determinants of Health; HR: Hazard ratio; CI: Confidence Interval; I^2^: Random effect; Ref: reference value.

All models were adjusted by age, sex, race, and cohort.

*: p < 0.05; **: p < 0.01; ***: p < 0.001

**S1 Fig. Participant’s study selection for HRS and ELSA populations.**


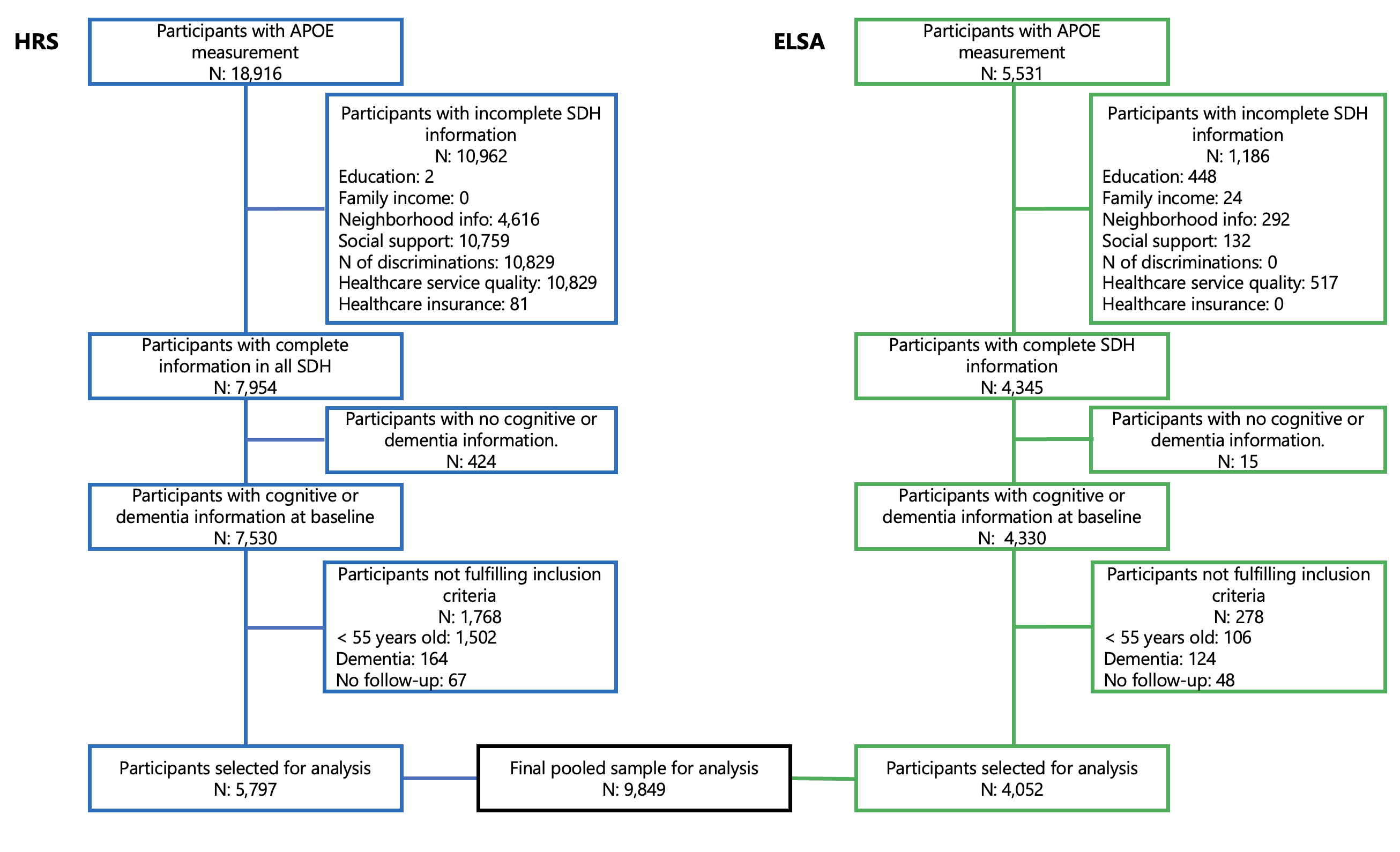


**S2 Fig. Social advantage Interacts with APOE Allele to Determine Risk of Developing Dementia.**

**
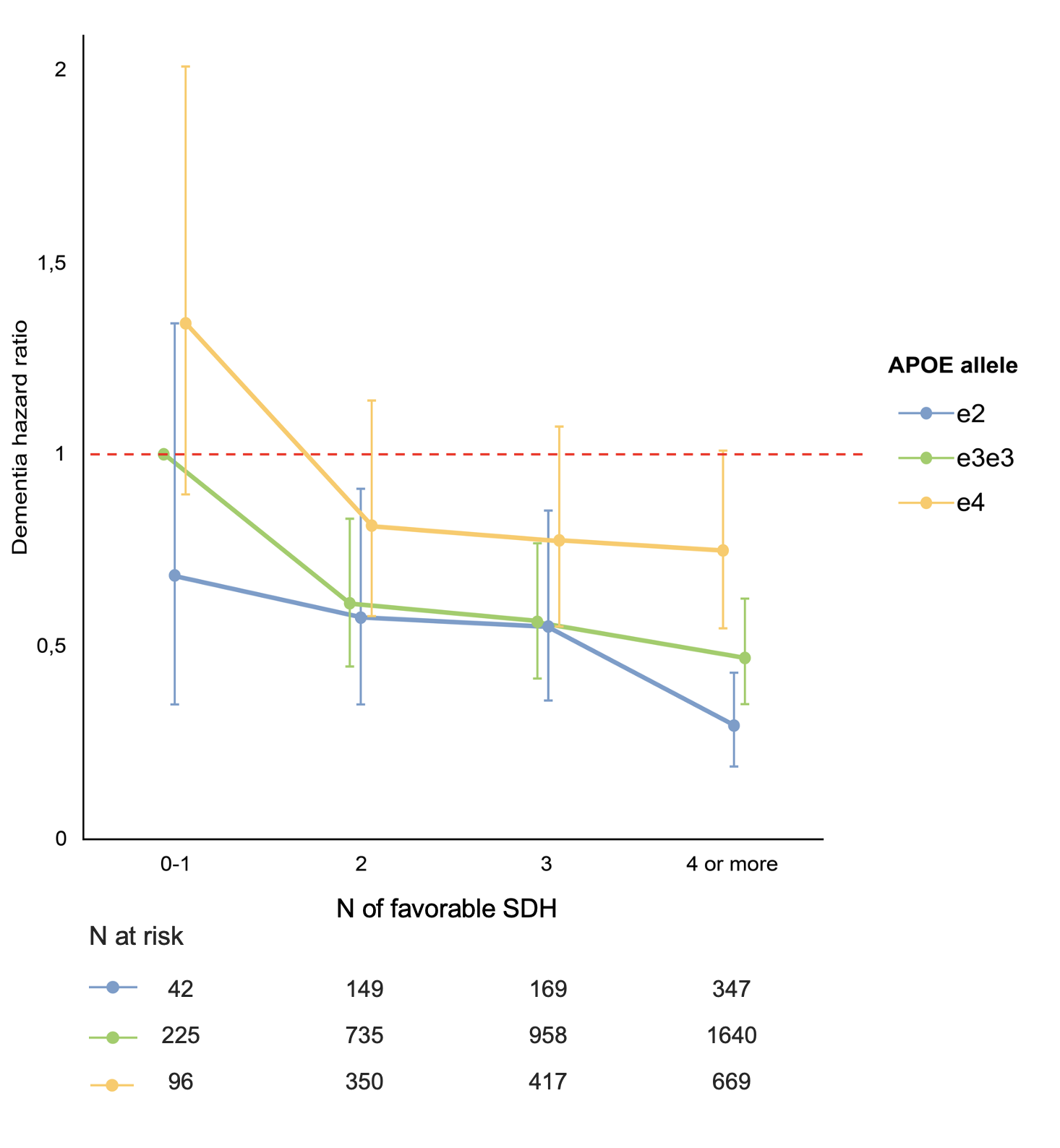
**

The figure describes the dementia Hazard Ratio by APOE allele profile and number of favorable social determinants of health (SDH) exposure compared to people at intermediate genetic risk (APOE-e3e3) exposed at 0-1 unfavorable SDH. APOE-e2: e2e2, e2e3; APOE-e4: e2e4, e3e4, e4e4.
