# Supplementary material for: Unequal expression: Social position modulates APOE genotype risk of dementia
Source: PLoS One. 2025 Nov 20;20(11):e0335846. doi: 10.1371/journal.pone.0335846 (PMC12633912; doi:10.1371/journal.pone.0335846)
Supplement: Attachment — Submitted filename: Response to reviewers.10.06.25.docx [file pone.0335846.s003.docx]

October 06^th^, 2025

Ioannis Liampas, MD, PhD

Academic Editor

*PLOS ONE*

RE: Manuscript ID PONE-D-25-31823 entitled: " Unequal Expression: Social Position Modulates APOE Genotype Risk of Dementia.”

Dear Dr. Liampas,

We appreciate the opportunity to address the editors and reviewers’ comments and revise our manuscript. We appreciate the chance to improve the formatting and results provided by our manuscript.

All page and paragraph numbers refer to locations in the revised manuscript.

As part of our response, we attach:

- Separate document uploaded as “Response to Reviewers” that addresses the issues raised in the below Executive Editor’s comments (with their helpful comments in bold and our responses not in bold).
- Revised Word document with track changes highlighting those changes in the manuscript itself entitled “Revised Manuscript with Track Changes.”
- Revised Word document without the changes highlighted (clean copy) entitled “Manuscript.”

**Role of Funder Statement**

The funders had no role in study design, data collection and analysis, decision to publish, or preparation of the manuscript.

Thank you again for your time and the opportunity to improve our manuscript.

Sincerely,

José M. Aravena, OT, MS, PhD

Department of Social & Behavioral Sciences

School of Public Health

Yale University.

Email: jose.aravena@yale.edu; phone: +(56)956583320.

**Journal Requirements:**

**When submitting your revision, we need you to address these additional requirements.**

**1.Please ensure that your manuscript meets PLOS ONE's style requirements, including those for file naming. The PLOS ONE style templates can be found at**

**https://journals.plos.org/plosone/s/file?id=wjVg/PLOSOne_formatting_sample_main_body.pdf and**

**https://journals.plos.org/plosone/s/file?id=ba62/PLOSOne_formatting_sample_title_authors_affiliations.pdf**

**Response:**

Thank you for the style clarification. We have edited all the manuscript according to both PLOS One style templates referenced.

**2. Please note that funding information should not appear in any section or other areas of your manuscript. We will only publish funding information present in the Funding Statement section of the online submission form. Please remove any funding-related text from the manuscript.**

**Response:**

Thank you for this observation. We have removed all funding-related text from the manuscript.

**3. We note that the grant information you provided in the ‘Funding Information’ and ‘Financial Disclosure’ sections do not match.**

**When you resubmit, please ensure that you provide the correct grant numbers for the awards you received for your study in the ‘Funding Information’ section.**

**Response:**

Thank you for this observation. We have corrected with the right information in the online Funding Statement section.

**4. Thank you for stating the following financial disclosure:**

**“The Health and Retirement Study (HRS) is supported by the National Institute on Aging (NIA U01AG009740) and the Social Security Administration funding the Health and Retirement Study. The English Longitudinal Study of Ageing (ELSA) is funded by the National Institute on Aging (Ref: R01AG017644) and by a consortium of UK government departments: Department for Health and Social Care; Department for Transport; Department for Work and Pensions, which is coordinated by the National Institute for Health Research (NIHR, Ref: 198-1074). Funding has also been provided by the Economic and Social Research Council (ESRC).**

**José M. Aravena was supported by a Fulbright Fellowship, a National Research and Development Agency of Chile (ANID) Fellowship, the Yale Social and Behavioral Sciences Research Fund, and grants from the Yale University Council on Latin American and Iberian Studies, and the Yale MacMillan Center for International and Area Studies. Xi Chen was supported by the National Institute on Aging grants R01AG077529 and P30AG021342. Becca R. Levy was supported by the National Institute on Aging grants R01AG067533 and U01AG032284.”**

**Please state what role the funders took in the study. If the funders had no role, please state: "The funders had no role in study design, data collection and analysis, decision to publish, or preparation of the manuscript."**

**If this statement is not correct you must amend it as needed.**

**Please include this amended Role of Funder statement in your cover letter; we will change the online submission form on your behalf.**

**Response:**

Thank you for this observation. We have included this statement in the introduction of the new cover letter.

**5. We note that you have indicated that there are restrictions to data sharing for this study. PLOS only allows data to be available upon request if there are legal or ethical restrictions on sharing data publicly. For more information on unacceptable data access restrictions, please see http://journals.plos.org/plosone/s/data-availability#loc-unacceptable-data-access-restrictions.**

**Before we proceed with your manuscript, please address the following prompts:**

**a) If there are ethical or legal restrictions on sharing a de-identified data set, please explain them in detail (e.g., data contain potentially identifying or sensitive patient information, data are owned by a third-party organization, etc.) and who has imposed them (e.g., a Research Ethics Committee or Institutional Review Board, etc.). Please also provide contact information for a data access committee, ethics committee, or other institutional body to which data requests may be sent.**

**b) If there are no restrictions, please upload the minimal anonymized data set necessary to replicate your study findings to a stable, public repository and provide us with the relevant URLs, DOIs, or accession numbers. For a list of recommended repositories, please see**

**https://journals.plos.org/plosone/s/recommended-repositories. You also have the option of uploading the data as Supporting Information files, but we would recommend depositing data directly to a data repository if possible.**

**We will update your Data Availability statement on your behalf to reflect the information you provide.**

**Response:**

We appreciate the opportunity to clarify our data availability statement. While the phenotypic and survey data from both HRS and ELSA are publicly accessible, access to genetic data is subject to legal and ethical restrictions imposed by the data custodians. Specifically, access to HRS genetic data is controlled by the University of Michigan and requires an application through the HRS data portal, due to the sensitive and potentially identifiable nature of genetic information. Similarly, access to ELSA genetic data is controlled by the ELSA project team at University College London and requires approval through a formal application process. These restrictions are in place to protect participant privacy and adhere to the ethical frameworks approved by the respective Institutional Review Boards.

Requests for HRS genetic data access can be submitted via the HRS data portal: <https://hrs.isr.umich.edu/data-products/genetic-data>.

Requests for ELSA genetic data access can be submitted via the ELSA project website: <https://www.elsa-project.ac.uk/genetic-data-access>

Additionally, we have modified in the manuscript the ‘Data availability’ statement accordingly (Page 12, Lines 264-271):

*“Phenotypic and survey data from the Health and Retirement Study (HRS) are publicly available via https://hrs.isr.umich.edu/data-products. Phenotypic data from the English Longitudinal Study of Ageing (ELSA) are available via https://ukdataservice.ac.uk. Access to genetic data from both HRS and ELSA is subject to legal and ethical restrictions due to the sensitive nature of genetic information. HRS genetic data can be requested via https://hrs.isr.umich.edu/data-products/genetic-data, and ELSA genetic data can be requested via https://www.elsa-project.ac.uk/genetic-data-access”*

**6. If the reviewer comments include a recommendation to cite specific previously published works, please review and evaluate these publications to determine whether they are relevant and should be cited. There is no requirement to cite these works unless the editor has indicated otherwise.**

**Response:**

Thank you for the clarification. No addition of citation recommendation was provided by reviewers.

**7. Please review your reference list to ensure that it is complete and correct. If you have cited papers that have been retracted, please include the rationale for doing so in the manuscript text, or remove these references and replace them with relevant current references. Any changes to the reference list should be mentioned in the rebuttal letter that accompanies your revised manuscript. If you need to cite a retracted article, indicate the article’s retracted status in the References list and also include a citation and full reference for the retraction notice.**

**Response:**

We thank the reviewers for this formatting reminder. We have carefully reviewed all references to ensure compliance with PLOS ONE's reference style, including the inclusion of DOIs where available. Additionally, we confirm that none of the cited articles have been retracted as of the time of submission.

**Review Comments to the Author**

**Reviewer #1: The manuscript is well-written and effectively outlines the interaction between environmental and genetic factors as contributors to Alzheimer’s disease (AD) risk among a diverse cohort. The methodology is clearly described and appropriately cited, and the results are presented in a structured and coherent manner. The discussion thoughtfully interprets the findings and is supported by relevant literature. The following are a few suggestions to enhance the manuscript further:**

**Response:**

We thank the reviewer for the comments and suggestions that have strengthen our manuscript.

**1.While Table 1 presents descriptive statistics stratified by APOE allele risk profile, it would be helpful to also include a summary of participant characteristics for the full sample, regardless of APOE status. This would provide a clearer overall picture of the study population.**

**Response:**

We thank the reviewer for this suggestion. We have edited and included a new Table 1 describing the characteristics of the overall sample (Pages 13-14, Lines 285-295):

**Table 1. Descriptive Statistics of the Sample at Baseline by APOE Allele Risk Profile.**

|  | **Total sample**  **n: 9849** | **Low risk**  **(APOE-ε2ε2, ε2ε3)**  **n: 1256** | **Intermediate risk**  **(APOE-ε3ε3)**  **n: 5968** | **High risk**  **(APOE-ε2ε4, ε3ε4, ε4ε4)**  **n: 2625** | **P-value** |
| --- | --- | --- | --- | --- | --- |
| Follow-up duration in years  Mean, SD (IQR) | 6.88, 3.792 (4.0 – 10.0) | 6.94, 3.867 (4.0 – 12.0) | 6.90, 3.817 (4.0 – 10.0) | 6.79, 3.702 (4.0 – 10.0) | 0.371 |
| Age  Mean, SD (IQR) | 68.45, 8.783 (62.0 – 74.0) | 68.64, 8.922 (62.0 – 75.0) | 68.68, 8.852 (62.0 – 75.0) | 67.82, 8.527 (61.0 – 73.0) | <0.001 |
| Sex n (%) |  |  |  |  |  |
| Male | 4404 (44.7) | 554 (44.1) | 2689 (45.1) | 1161 (44.2) | 0.698 |
| Female | 5445 (55.3) | 702 (55.9) | 3279 (54.9) | 1464 (55.8) |  |
| Race/ethnicity, n (%) |  |  |  |  |  |
| Non-Hispanic White | 8832 (89.7) | 1132 (90.1) | 5406 (90.6) | 2294 (87.4) | <0.001 |
| Non-Hispanic Black | 766 (7.8) | 104 (8.3) | 394 (6.6) | 268 (10.2) |  |
| Other (American  Indian, Alaskan  Native, Asian, and  Pacific Islander) | 251 (2.5) | 20 (1.6) | 168 (2.8) | 63 (2.4) |  |
| Hispanic | 454 (4.6) | 30 (4.2) | 320 (9.0) | 104 (6.8) |  |
| Dementia risk factors, n (%) |  |  |  |  |  |
| Diabetes | 1477 (15.0) | 185 (14.7) | 921 (15.4) | 371 (14.1) | 0.287 |
| Hypertension | 4954 (50.3) | 616 (49.0) | 3008 (50.4) | 1330 (50.7) | 0.619 |
| Obesity | 2959 (31.5) | 387 (32.2) | 1825 (32.0) | 747 (29.8) | 0.122 |
| High-frequency drinking | 1274 (13.0) | 166 (13.3) | 770 (13.0) | 338 (13.0) | 0.947 |
| Physical inactivity | 2252 (22.9) | 291 (23.2) | 1378 (23.1) | 583 (22.2) | 0.652 |
| Smoking | 1150 (11.7) | 150 (12.0) | 680 (11.4) | 320 (12.2) | 0.536 |
| Hearing problems | 435 (4.4) | 53 (4.2) | 273 (4.6) | 109 (4.2) | 0.635 |
| Depression | 1836 (18.6) | 231 (18.4) | 1127 (18.9) | 478 (18.2) | 0.729 |
| N of dementia risk factors, n (%) |  |  |  |  |  |
| 0 | 1797 (18.2) | 232 (18.5) | 1086 (18.2) | 479 (18.2) | 0.977 |
| 1 | 3131 (31.8) | 395 (31.4) | 1888 (31.6) | 848 (32.3) |  |
| 2 | 2512 (25.5) | 323 (25.7) | 1517 (25.4) | 672 (25.6) |  |
| ≥3 | 2409 (24.5) | 306 (24.3) | 1475 (24.7) | 626 (23.8) |  |
| Social determinants of health |  |  |  |  |  |
| Education level, n (%) |  |  |  |  |  |
| Less than upper secondary | 2066 (21.0) | 247 (19.7) | 1236 (20.7) | 583 (22.2) | 0.209 |
| Upper secondary and vocational | 5571 (56.6) | 704 (56.1) | 3398 (56.9) | 1469 (56.0) |  |
| Tertiary | 2212 (22.5) | 305 (24.3) | 1334 (22.4) | 573 (21.8) |  |
| Family income quartile by cohort, n (%) |  |  |  |  |  |
| <25^th^ quartile income | 2132 (21.6) | 268 (21.3) | 1296 (21.7) | 568 (21.6) | 0.998 |
| 25^th^ – 75^th^ quartile income | 5120 (52.0) | 656 (52.2) | 3102 (52.0) | 1362 (51.9) |  |
| >75^th^ quartile income | 2597 (26.4) | 332 (26.4) | 1570 (26.3) | 695 (26.5) |  |
| Healthcare access, n (%) |  |  |  |  |  |
| No private health insurance | 4776 (48.5) | 617 (49.1) | 2932 (49.1) | 1227 (46.7) | 0.112 |
| Healthcare discrimination | 546 (5.5) | 67 (5.3) | 331 (5.5) | 148 (5.6) | 0.928 |
| Neighborhood environment, n (%) |  |  |  |  |  |
| Physical disorder |  |  |  |  |  |
| Low (<25^th^) | 2071 (21.0) | 248 (19.7) | 1242 (20.8) | 581 (22.1) | 0.331 |
| Moderate (25^th^ – 75^th^) | 4614 (46.8) | 585 (46.6) | 2800 (46.9) | 1229 (46.9) |  |
| High (>75^th^) | 3164 (32.2) | 423 (33.7) | 1926 (32.3) | 815 (31.0) |  |
| Social cohesion difficulties |  |  |  |  |  |
| Low (<25^th^) | 2079 (21.1) | 267 (21.3) | 1232 (20.6) | 580 (22.1) | 0.247 |
| Moderate (25^th^ – 75^th^) | 4640 (47.1) | 568 (45.2) | 2829 (47.4) | 1243 (47.4) |  |
| High (>75^th^) | 3130 (31.8) | 421 (33.5) | 1907 (32.0) | 802 (30.6) |  |
| Social and community context, n (%) |  |  |  |  |  |
| Social support |  |  |  |  |  |
| Low (<25^th^) | 2221 (22.6) | 259 (20.6) | 1357 (22.7) | 605 (23.0) | 0.009 |
| Moderate (25^th^ – 75^th^) | 5097 (51.8) | 629 (50.1) | 3076 (51.5) | 1392 (53.0) |  |
| High (>75^th^) | 2531 (25.7) | 368 (29.3) | 1535 (25.7) | 628 (23.9) |  |
| N of types of discrimination |  |  |  |  |  |
| 0 | 4692 (47.6) | 605 (48.2) | 2850 (47.8) | 1237 (47.1) | 0.791 |
| 1 | 3358 (34.1) | 432 (34.4) | 2012 (33.7) | 914 (34.8) |  |
| ≥2 | 1799 (18.3) | 219 (17.4) | 1106 (18.5) | 474 (18.1) |  |
| Social adversity: n of unfavorable SDH |  |  |  |  |  |
| ≤ 1 | 4201 (42.7) | 571 (45.5) | 2549 (42.7) | 1081 (41.2) | 0.143 |
| 2 | 2854 (29.0) | 344 (27.4) | 1739 (29.1) | 771 (29.4) |  |
| 3 | 1666 (16.9) | 190 (15.1) | 1004 (16.8) | 472 (18.0) |  |
| ≥ 4 | 1128 (11.4) | 151 (12.0) | 676 (11.3) | 301 (11.5) |  |
| Any functional limitations in ADL, n (%) | 1340 (13.6) | 169 (13.5) | 826 (13.8) | 345 (13.1) | 0.676 |

SDH: Social Determinants of Health; IQR: interquartile range; ADL: Activities of daily living.

<25^th^: less than 25^th^ quartile by cohort; 25^th^ – 75^th^: between 25^th^ and 75^th^ quartile by cohort; >75^th^: more than 75^th^ quartile by cohort.

Social adversity measured as N of unfavorable social determinants of health (SDH) exposed: Less than upper secondary education, being in the 25^th^ lower family income range, 25^th^ lower score in neighborhood physical disorder, 25^th^ lower score in neighborhood social cohesion, having experienced poorer health service from a doctor or hospital, not having some private healthcare insurance, 25^th^ lower score in perceived social support, having experienced two or more different types of discrimination.

P-value for chi-square test (X^2^) for nominal and ordinal variables and ANOVA test for continuous variables comparison.

**2. Author should also explore whether the interaction between APOE genotype and social adversity differs by sex or racial/ethnic group.**

**Response:**

We thank the reviewer for this suggestion. We have included a section to describe the interaction between APOE genotype and social adversity by sex (female and male) and racial group (Black and White people). These results are described as part of the statistical analysis (Page 12), provided in detail in the supplementary material (Table S9 for sex differences and Table S10 for race differences), and summarized in the manuscript’ results (Page 23 and 24, Lines 458-474):

*“Subgroup analysis by sex showed that, in both males and females, the same pattern of APOE allele impact was observed: the effect was more evident among individuals living with low social adversity, whereas among those exposed to greater social adversity, the influence of the APOE allele was less pronounced (Table S9.a). However, the impact of social adversity on APOE genotype subgroups was more evident in women. We also observed that the effect of social adversity on male APOE-ε2 carriers was less pronounced than in female carriers, whereas the opposite pattern was found for APOE-ε4, with a stronger effect among female carriers compared to male carriers (Table S9.b).*

*Regarding racial differences (Black and White individuals), the same effect was observed: people at high social disadvantage presented the greatest risk of dementia, regardless of APOE allele (Table S10.a). Among White participants, APOE-ε2 carriers at social advantage presented a lower risk of dementia, and APOE-ε4 carriers at social advantage had a greater risk of dementia than APOE-ε3ε3 carriers at social advantage (Table S10.b). In contrast, among Black participants, APOE-ε2 carriers at social advantage did not present a lower risk of dementia, and APOE-ε4 carriers at social advantage showed no difference in dementia risk compared to APOE-ε3ε3 carriers at social advantage (Table S10.b).”*

**3. Since social adversity was constructed using five domains of social determinants of health (SDH), it would be informative to identify which specific domain(s) demonstrated the strongest interaction with APOE genotype in relation to dementia risk.**

**Response:**

We thank the reviewer for this suggestion. We have included a section to describe the impact of individual social determinants on dementia risk by APOE allele group. These results are described as part of the statistical analysis (Page 12); a detailed description of the results are provided in the supplementary material (Table S11.a to S11.c) and summarized in the manuscript (Page 24, Lines 475-487):

*“Regarding individual social determinants, having a lower education level and lower family income presented the strongest interactions and were associated with a greater risk of dementia, regardless of APOE allele group (Table S11.a). Interestingly, among APOE-ε4 carriers, only having less than upper secondary education was associated with greater dementia risk, whereas dementia risk remained elevated across all income levels (intermediate and low) when compared to those with high family income (Table S11.a).*

*In terms of healthcare-related factors, lower access to and quality of healthcare were associated with increased dementia risk among APOE-ε3ε3 and APOE-ε4 carriers (Table S11.b). Meanwhile, lower perceived social support and higher levels of neighborhood physical disorder were linked to greater dementia risk among APOE-ε2 and APOE-ε3ε3 carriers (Table S11.b and 11.c). Finally, lower neighborhood social cohesion was associated with increased dementia risk, but only among APOE-ε3ε3 carriers (Table S11.c).”*

**4. In addition to the limitations already outlined, add the unique strengths and contributions of this study.**

**Response:**

We thank the reviewer for acknowledging the strength and uniqueness of our study. We have enriched and added the following paragraphs after the limitation section (Page 28, Lines 569-588):

*“Our study has several strengths. We used two of the largest and most rigorously harmonized longitudinal cohorts of aging, HRS and ELSA, which enabled prospective analysis of dementia across diverse national settings. We applied a comprehensive, multidimensional operationalization of social determinants of health (SDH) based on the Healthy People 2030 framework,(20) capturing economic, educational, healthcare, social, and environmental dimensions. Unlike most prior studies, we included all APOE genotypes, allowing for nuanced analyses of gene–environment interactions. Dementia diagnoses were harmonized using validated algorithms across cohorts, enhancing comparability. Finally, we conducted six complementary sensitivity analyses, reinforcing the internal validity and robustness of our findings.*

*Beyond these methodological strengths, this study makes a unique contribution by being the first to robustly test the social distinction model of gene–environment interaction in the context of dementia. Unlike prior research that has focused on additive effects of genes and environment, our findings demonstrate how social advantage and disadvantage modify the expression of genetic risk, revealing that genetic risk may be less detectable, and potentially less actionable, in contexts of structural adversity. These insights help explain disparities in dementia incidence across social groups and suggest that the predictive value of genetic information is not universal, but conditional on societal context. This novel approach deepens our understanding of how macro-level factors shape biological vulnerability and offers a compelling case for incorporating SDH into precision brain health efforts.”*

**Reviewer #2: The authors demonstrated that while genetic factors, particularly APOE status, are central to dementia risk prediction, their impact is strongly moderated by social position. Using data from two large longitudinal cohorts (HRS and ELSA), they constructed a social adversity index based on education, economic stability, healthcare, neighborhood, and social context. Their analyses revealed that genetic risk was most evident among individuals with social advantage, whereas high social adversity elevated dementia risk across all genotypes. Notably, individuals with high genetic risk but social advantage had a lower dementia risk than those with low genetic risk but high social adversity. These findings highlight that social environments substantially shape the effect of genetic risk, emphasizing the importance of addressing social adversity to reduce dementia risk and improve equity in prevention strategies. Overall, the authors did an excellent job presenting their study. The manuscript is well written, highly readable, and demonstrates careful scientific consideration. The integration of genetic and social determinants of health is both timely and impactful, making this a strong contribution to the dementia prevention literature.**

**Response:** We thank the reviewers’ comments acknowledging the strength of our study.

**Reviewer #3: The manuscript “Unequal Expression: Social Position Modulates APOE Genotype Risk of Dementia” demonstrates that genetic risks affect mostly those with social advantage, and those with high social adversity have higher risks for dementia regardless of their genetic risks. The manuscript is well-written, the aims are clear, and it’s well-presented with acceptable flow. The idea in this manuscript is novel, and the data adds knowledge to the field. I do not have much, but the following comment for the improvement of the manuscript: The resolution for figures 1-3 is very low.**

**Response:** We thank the reviewers’ comments acknowledging the strength of our study. We have included figures in a better resolution following *PLOS One* guidelines.
